# Supplementary material for: Inhibition of ACSS2-mediated histone crotonylation alleviates kidney fibrosis via IL-1β-dependent macrophage activation and tubular cell senescence
Source: Nat Commun. 2024 Apr 13;15:3200. doi: 10.1038/s41467-024-47315-3 (PMC11016098; doi:10.1038/s41467-024-47315-3)
Supplement: Supplementary file 1 — Supplementary Information [file 41467_2024_47315_MOESM1_ESM.pdf]

Supplementary figures

Supplementary Fig. 1. The increased levels of crotonylation in human renal biopsies of CKD and fibrotic kidneys from mice. (A) Representative IHC staining of pan-Kcr antibody in healthy control subjects and CKD patients focused on glomerular. Scale bar: 20 μm. (n= 3 per group). (B) Quantitative IHC analysis of pan-Kcr expression in renal cytoplasm from control and CKD patients using ImageJ 6.0 software. (C) IF staining of pan-Kcr (green) and DAPI (blue) in control and UUO kidneys. Scale bar: 50 μm. (n= 3 per group). (D) Protein expression and quantitative analysis of histone crotonylation in the fibrotic kidneys of mice. (E) Coomassie brilliant blue staining of histone in the fibrotic kidneys of mice. (F) Quantitative analysis of H3K9cr, H3K9ac and H3 in the fibrotic kidneys of UUO mice. (G) Representative IHC staining and quantitative analysis of H3k9cr in healthy control subjects and CKD patients. Scale bar: 50 μm. (n= 3 per group). (H) Protein expression of H3K9cr, H3K9ac and H3 in the fibrotic kidneys of FAN mice were determined by western blotting (n = 6 animals per group). (I) Quantitative analysis of H3K9cr, H3K9ac and H3 in the fibrotic kidneys of FA mice. IHC: immunohistochemical; pan-Kcr: pan anti-crotonyllysine; IF: Immunofluorescence; FAN: folic acid nephropathy; UUO: unilateral ureteric obstruction. Triangle: representative positive staining of pan-Kcr. Data shown are means ± SEM. Statistical analysis by one-way ANOVA with Tukey's post hoc test. *$P < 0.05$.

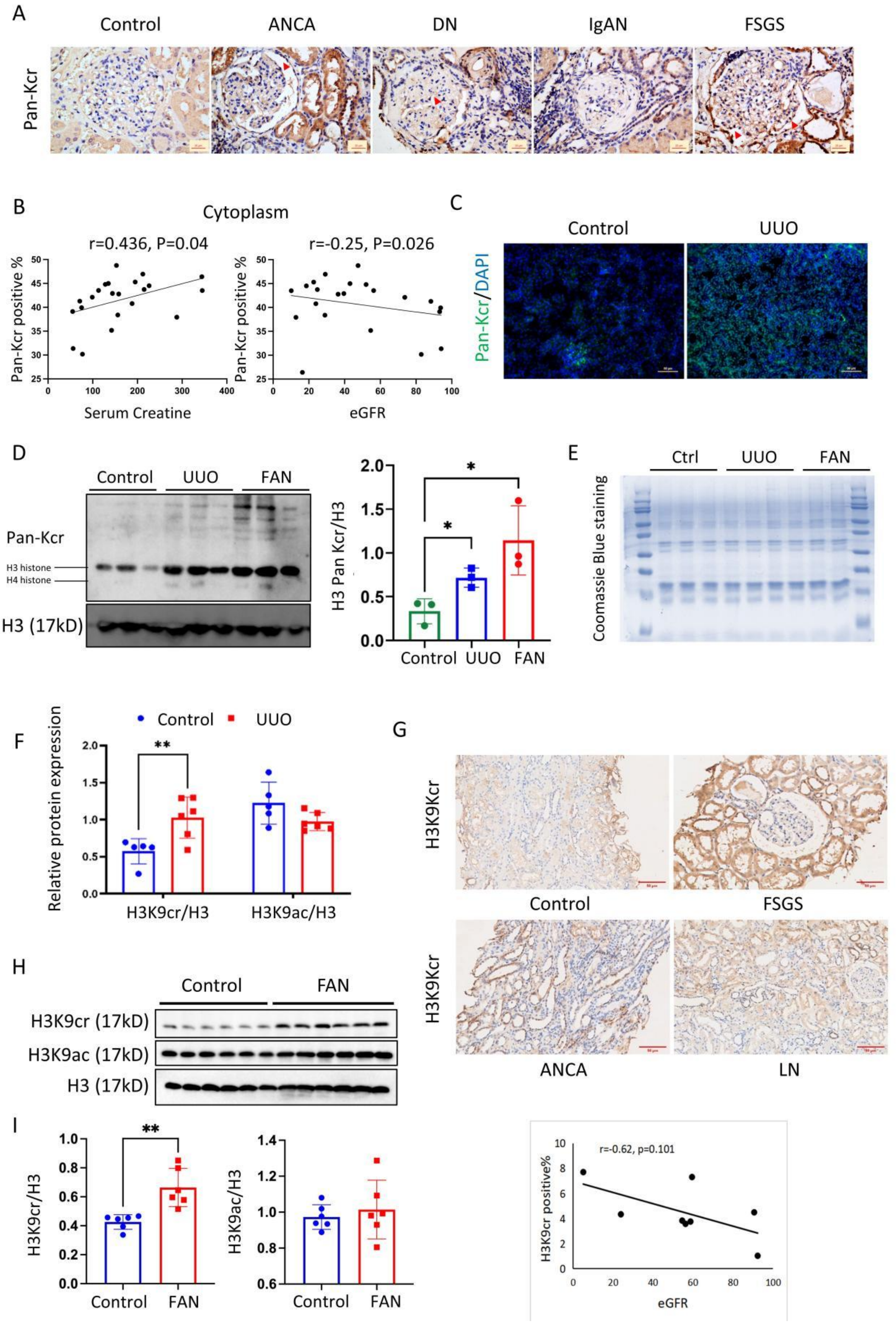
A
Control
ANCA
DN
IgAN
FSGS
Pan-Kcr
B
Cytoplasm
r=0.436, P=0.04
r=-0.25, P=0.026
Pan-Kcr positive %
Serum Creatine
eGFR
C
Control
UUO
Pan-Kcr/DAPI
D
Control
UUO
FAN
Pan-Kcr
H3 histone
H4 histone
H3 (17kD)
H3 Pan Kcr/H3
E
Ctrl
UUO
FAN
Coomassie Blue staining
F
Control
UUO
Relative protein expression
H3K9cr/H3
H3K9ac/H3
G
H3K9Kcr
Control
FSGS
ANCA
LN
H
Control
FAN
H3K9cr (17kD)
H3K9ac (17kD)
H3 (17kD)
I
H3K9cr/H3
H3K9ac/H3
Control
FAN
r=-0.62, p=0.101
H3K9cr positive%
eGFR

Supplementary Fig. 2. Changes of other lysine crotonylation and acetylation in fibrotic kidneys of mice. (A) Coomassie brilliant blue used for staining of gels to demonstrate histone purification. (B-C) subcellar classify and COG classify of histone LC/MS analysis. (D) Representative images of H&E and Masson staining in control and fibrotic mice kidneys. Scale bar: 50 μm.(n= 6 per group). (E) mRNA expression of Fn1, Col1a1 and Acta2 in control and fibrotic mice kidneys was determined by real-time qPCR (n = 3 to 6 animals per group). (F-G) Protein expression of FN1, COL6 and a-SMA in the fibrotic kidneys of mice was determined by western blotting (n = 5 to 6 animals per group). (H-K) Protein expression of H3K14cr, H3K18cr, H3K27cr, and H3K14ac, H3K18ac, H3K27ac in the fibrotic kidneys of mice were determined by western blotting (n = 5 to 6 animals per group). FN1: fibronectin; COL6: collagen type6; α-SMA: smooth muscle actin; FAN: folic acid nephropathy; UUO: unilateral ureteric obstruction. Data shown are means ± SEM. Statistical analysis by t-test. *P < 0.05, **P < 0.01, ***P < 0.001 and ****P < 0.0001.

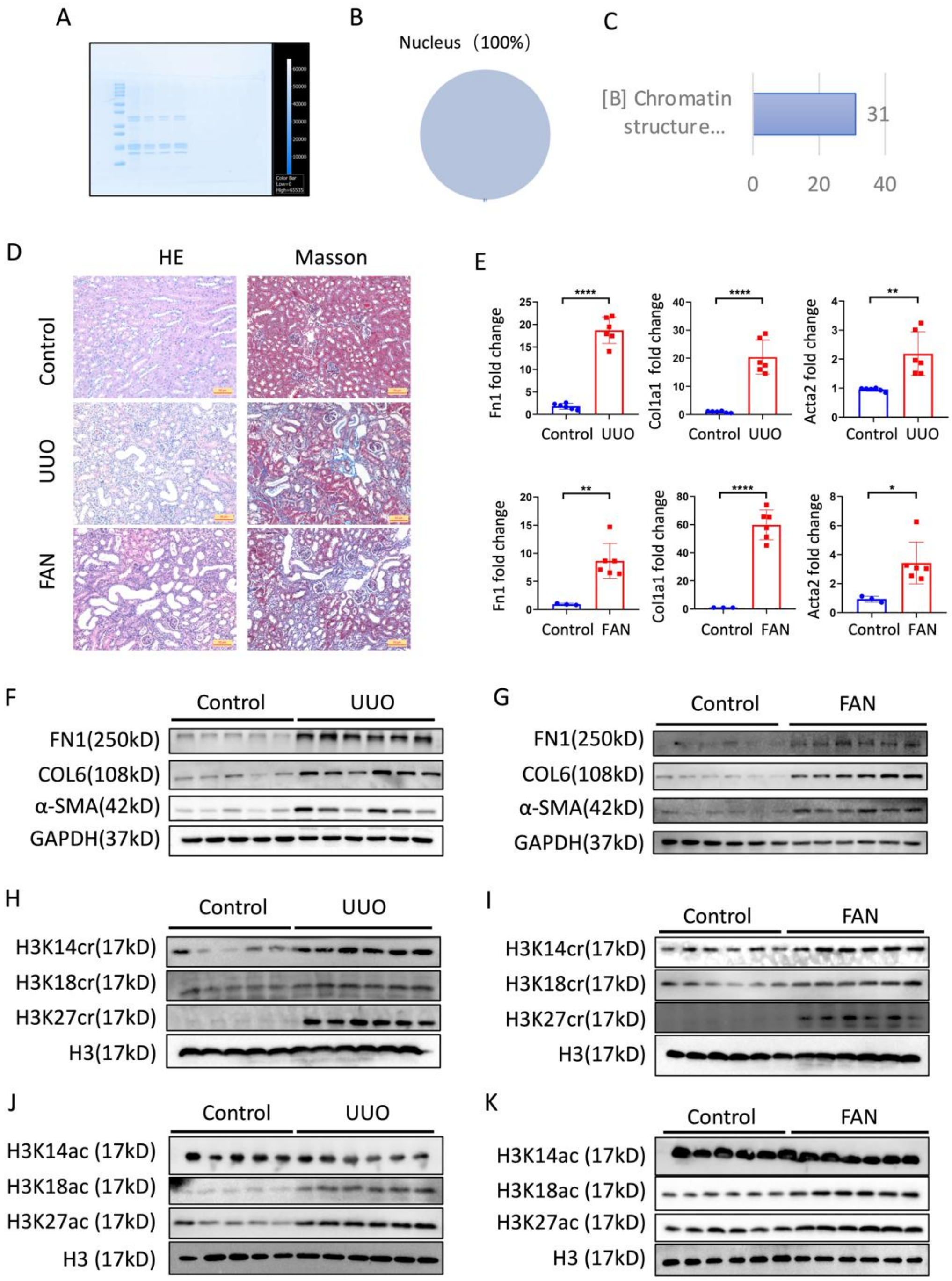
A
B
Nucleus（100%）
C
[B] Chromatin structure...
31
0
20
40
D
HE
Masson
Control
UUO
FAN
E
Fn1 fold change
Col1a1 fold change
Acta2 fold change
Control
UUO
FAN
****
**
*
F
G
Control
UUO
FAN
FN1(250kD)
COL6(108kD)
α-SMA(42kD)
GAPDH(37kD)
H
I
H3K14cr(17kD)
H3K18cr(17kD)
H3K27cr(17kD)
H3(17kD)
J
K
H3K14ac (17kD)
H3K18ac (17kD)
H3K27ac (17kD)
H3 (17kD)

Supplementary Fig. 3. Basic information of TCMK-1 and HEK-293T cells treated with crotonate or plasmids. (A) Protein and mRNA expression of ACSS2 in TCMK-1 cells treated with ACSS2 plasmids. (B) Protein and mRNA expression of ACSS2 in HEK-293T cells treated with ACSS2 plasmids. (C) Protein and mRNA expression of ACSS2 in TCMK-1 cells treated with 10 μM crotonate. (D) Protein and mRNA expression of ACSS2 in HEK-293T cells treated with 10 μM crotonate. (E-F) Protein and mRNA expression of SIRT1/2/3 in TCMK-1 cells treated with SIRT1/2/3 plasmids. (G-H) Protein and mRNA expression of SIRT1/2/3 in HEK-293T cells treated with SIRT1/2/3 plasmids. (I-J) Protein and mRNA expression of HDAC1/2/3 in TCMK-1 cells treated with HDAC1/2/3 plasmids. (K-L) Protein and mRNA expression of HDAC1/2/3 in HEK-293T cells treated with HDAC1/2/3 plasmids. (M-N) Protein and mRNA expression of SIRT4/5/6 in TCMK-1 cells treated with SIRT4/5/6 plasmids. (O-P) Protein and mRNA expression of SIRT4/5/6 in HEK-293T cells treated with SIRT4/5/6 plasmids. CR10: 10 μM crotonate; SIRT1/2/3/4/5/6 OE: SIRT1/2/3/4/5/6 overexpression; HDAC1/2/3 OE: HDAC1/2/3 overexpression. Data shown are means ± SEM. Statistical analysis by t-test. $^{*}P < 0.05$, $^{**}P < 0.01$, $^{***}P < 0.001$ and $^{****}P < 0.0001$.

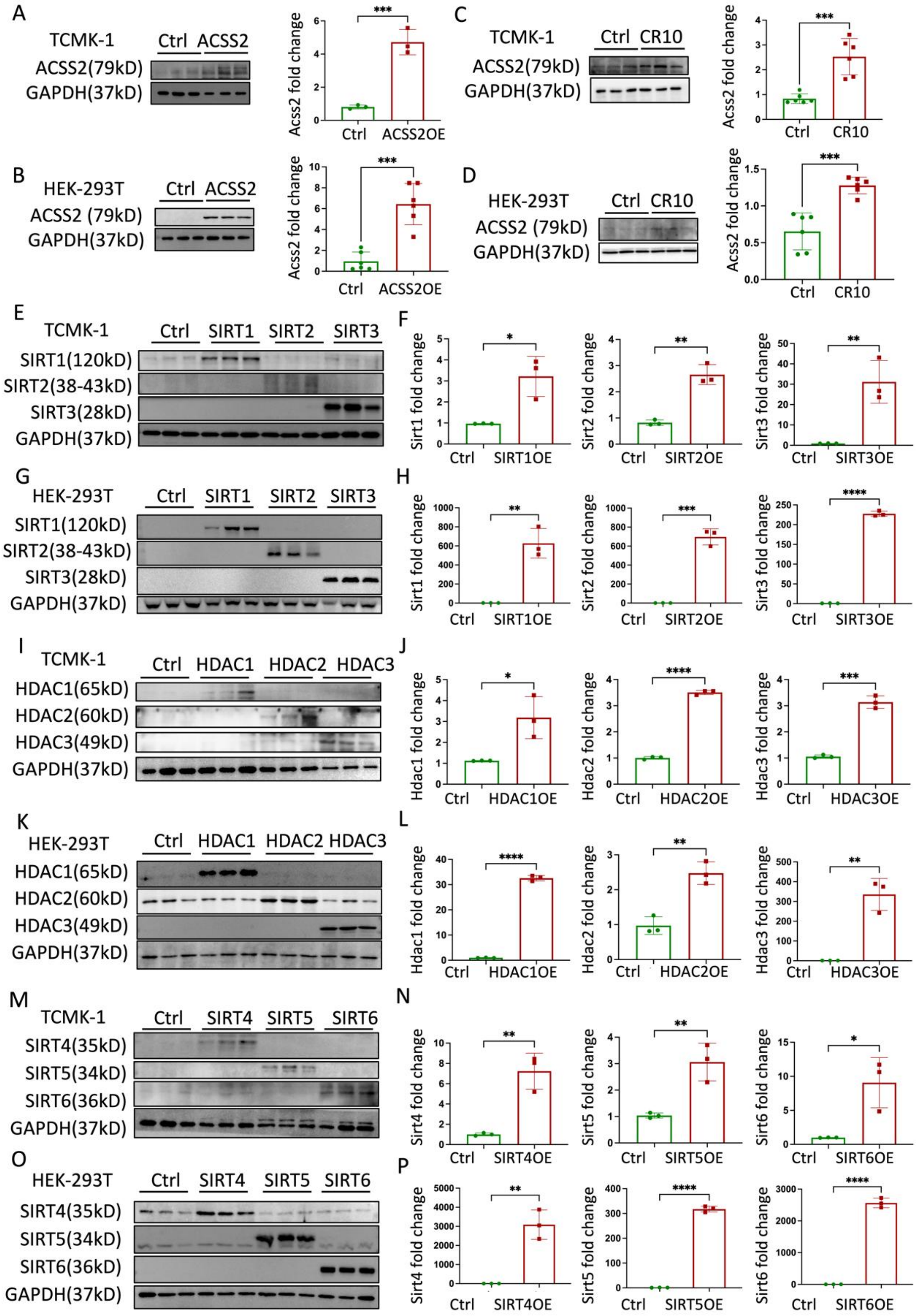
A
TCMK-1
Ctrl ACSS2
ACSS2(79kD)
GAPDH(37kD)
Acss2 fold change
Ctrl ACSS2OE
B
HEK-293T
ACSS2 (79kD)
C
CR10
D
E
SIRT1(120kD)
SIRT2(38-43kD)
SIRT3(28kD)
F
G
H
I
HDAC1(65kD)
HDAC2(60kD)
HDAC3(49kD)
J
K
L
M
SIRT4(35kD)
SIRT5(34kD)
SIRT6(36kD)
N
O
P

Supplementary Fig. 4. The changes of H3K9cr and H3K9ac level under different treatment *in vitro*. (A) Schematic illustration of different treatment for H3K9cr and H3K9ac in cells. (B) Protein expression and quantitative analysis of H3K9cr, H3K9ac and H3 when overexpression of ACSS2 using relative plasmids in TCMK-1 cells and HEK-293T cells (n = 3 per group). (C-D) Protein expression and quantitative analysis of H3K9cr, H3K9ac and H3 when treated with 10 μM crotonate in TCMK-1 cells and HEK-293T cells (n = 3 per group). (E-F) Protein level and quantitative analysis of H3K9cr, H3K9ac and H3 when treated with HDAC1/2/3 plasmids in TCMK-1 cells and HEK-293T cells (n = 3 per group). (G-H) Protein level and quantitative analysis of H3K9cr, H3K9ac and H3 when treated with SIRT1/2/3 plasmids in TCMK-1 cells and HEK-293T cells (n = 3 per group). (I-J) Protein level and quantitative analysis of H3K9cr, H3K9ac and H3 when treated with SIRT4/5/6 plasmids in TCMK-1 cells and HEK-293T cells (n = 3 per group). CR10: 10 μM crotonate. CTRL: Control. Data shown are means ± SEM. Statistical analysis by t-test or one-way ANOVA with Tukey's post hoc test. *P < 0.05, **P < 0.01, ***P < 0.001 and ****P < 0.0001 versus the control.

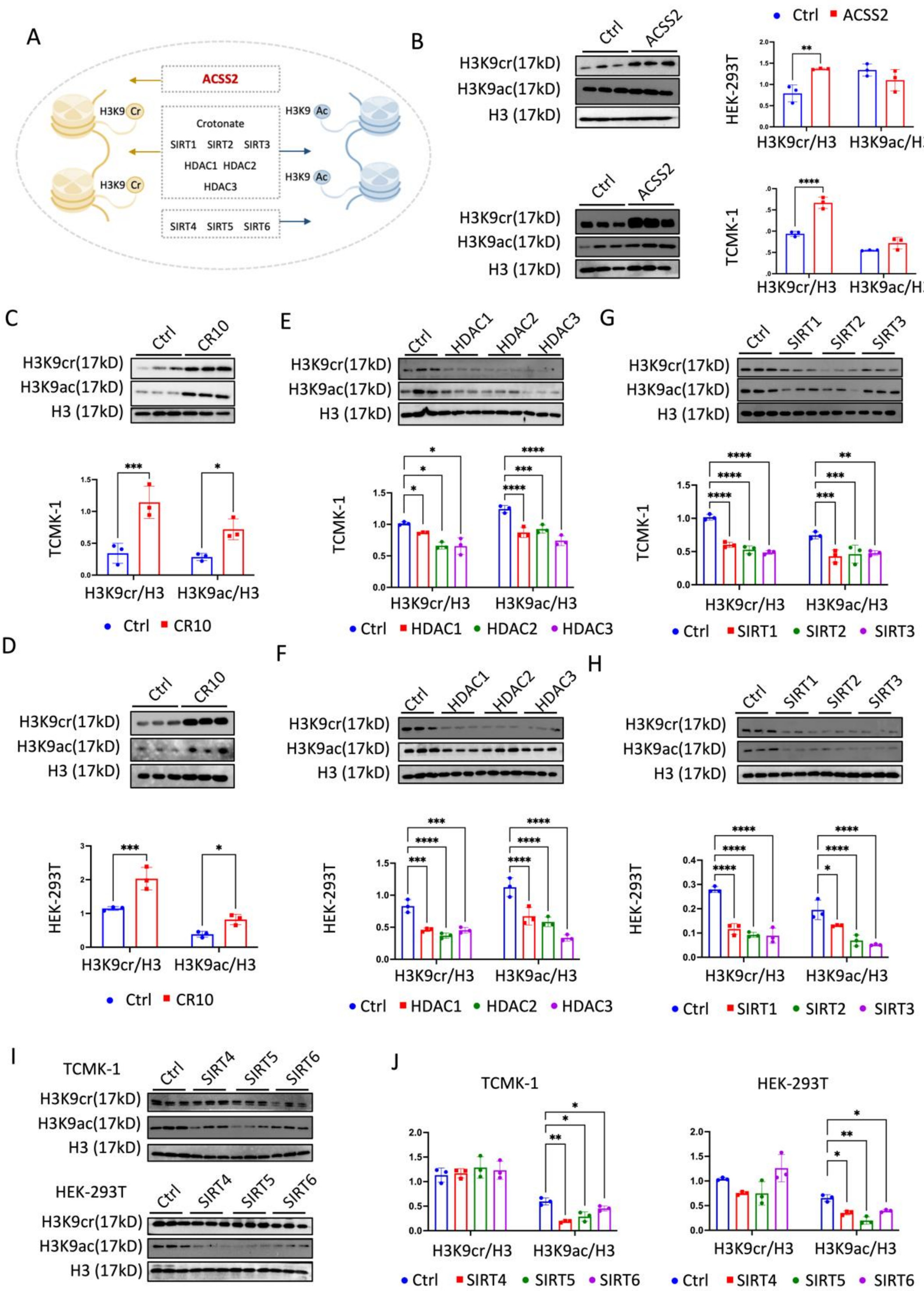
A
ACSS2
H3K9 Cr
Crotonate
SIRT1 SIRT2 SIRT3
HDAC1 HDAC2
HDAC3
H3K9 Ac
SIRT4 SIRT5 SIRT6
B
Ctrl
ACSS2
H3K9cr(17kD)
H3K9ac(17kD)
H3 (17kD)
HEK-293T
TCMK-1
H3K9cr/H3
H3K9ac/H3
C
CR10
D
E
HDAC1
HDAC2
HDAC3
F
G
SIRT1
SIRT2
SIRT3
H
I
SIRT4
SIRT5
SIRT6
J

Supplementary Fig. 5. Generation of ACSS2$^{-/-}$ mice. (A) Schematic generation of ACSS2$^{-/-}$ mice. (B-C) Identification of the genotype of ACSS2$^{-/-}$ mice by PCR assay. (D) Protein expression of ACSS2 and GAPDH in whole kidney lysates of control and UUO of WT and ACSS2$^{-/-}$ mice (n = 3 per group). (E) Protein expression of ACSS2 and GAPDH in whole kidney lysates of control and FAN of WT and ACSS2$^{-/-}$ mice (n = 3 per group). FAN: folic acid nephropathy; UUO: unilateral ureteric obstruction; WT: wild type; ACSS2$^{-/-}$: ACSS2 knockout mice.

A

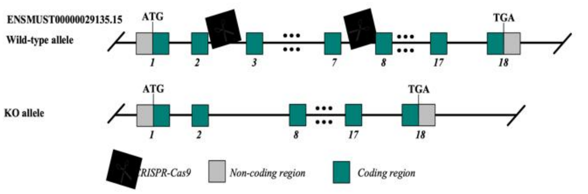

B

	Primer Name	Sequence (5'-3')	PCR size
1	JS05949-Acss2-5wt-tF1	TCACTTGAGAACTTCCTACCTTAGCC	(WT: 4769bp) -4454bp=315bp
	JS05949-Acss2-3wt-tR1	GGAAGCAGAATGAGCTGTTAGTGAAAC	
2	JS15949-Acss2-wt-tF1	AAAGCTGTGAAGGTTGACATGCAC	WT: 333bp
	JS15949-Acss2-wt-tR1	ATATTGATTGGGCTCCCCAGTCT	

C

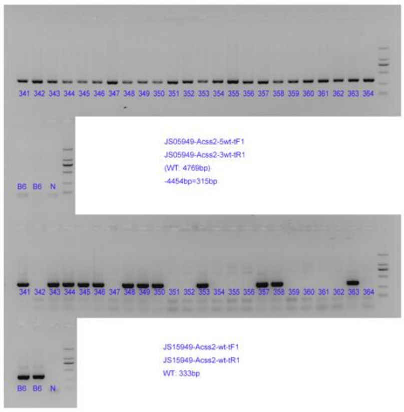

P: Positive Control ; B6: Negative Control; N: blank

DL2000 Marker：2000bp\1000bp\750bp\500bp\250bp\100bp

Acss2 -4454bp/-4454bp：342，347，351，352，354-356，359-362，364

D

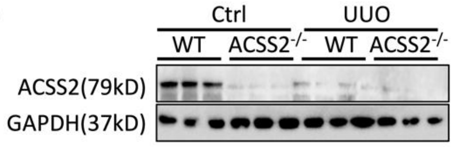

E

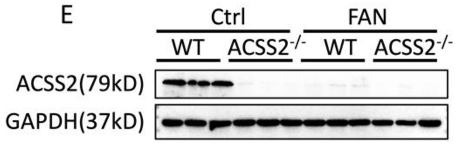

Supplementary Fig. 6. The **scRNA-seq and spatial transcription analysis.** (A) The expression of ACSS2 in proximal tubule cell of health and IRI/UUO mice in scRNA-seq. (B) The expression of ACSS2 in proximal tubule cell of health and CKD patients in scRNA-seq. (C) Representative IHC staining of ACSS2 in medulla, corticomedullary junction and cortex of mice fibrotic kidney. Scale bar: 100 μm. (n= 3 per group). (D) UMAP plots of expression of the marker genes for PTs12 and PTs3 in mice and CKD in scRNA-seq.(E) UMAP plots of expression of the marker genes for PTs12 and PTs3 in mice in spatial transcription analysis. (F) UMAPs of cell type clustering in kidneys. Cell types include proximal tubule segments 1-2 (PT S1-S2), proximal tubule segment 3 (PT S3), distal convoluted tubules (DCT), intercalated cells (IC), podocytes (Pod), fibroblasts (Fib), thick ascending limb (TAL), principal cells (PC), urothelium (Uro), and proximal tubule segment 3- thick ascending limb (PT S3-TAL), macrophage (Macro), adipocytes (Adipo). (G) Heatmap showing the expression patterns of the top ten marker genes in each cell cluster. (H) The expression of ACSS2 in health and IRI mice in spatial transcription. IRI: ischemia reperfusion injury; Sham: Sham surgery mice; CKD: chronic kidney disease; UUO: unilateral ureteric obstruction; 6we: 6 weeks after ischemia reperfusion injury.

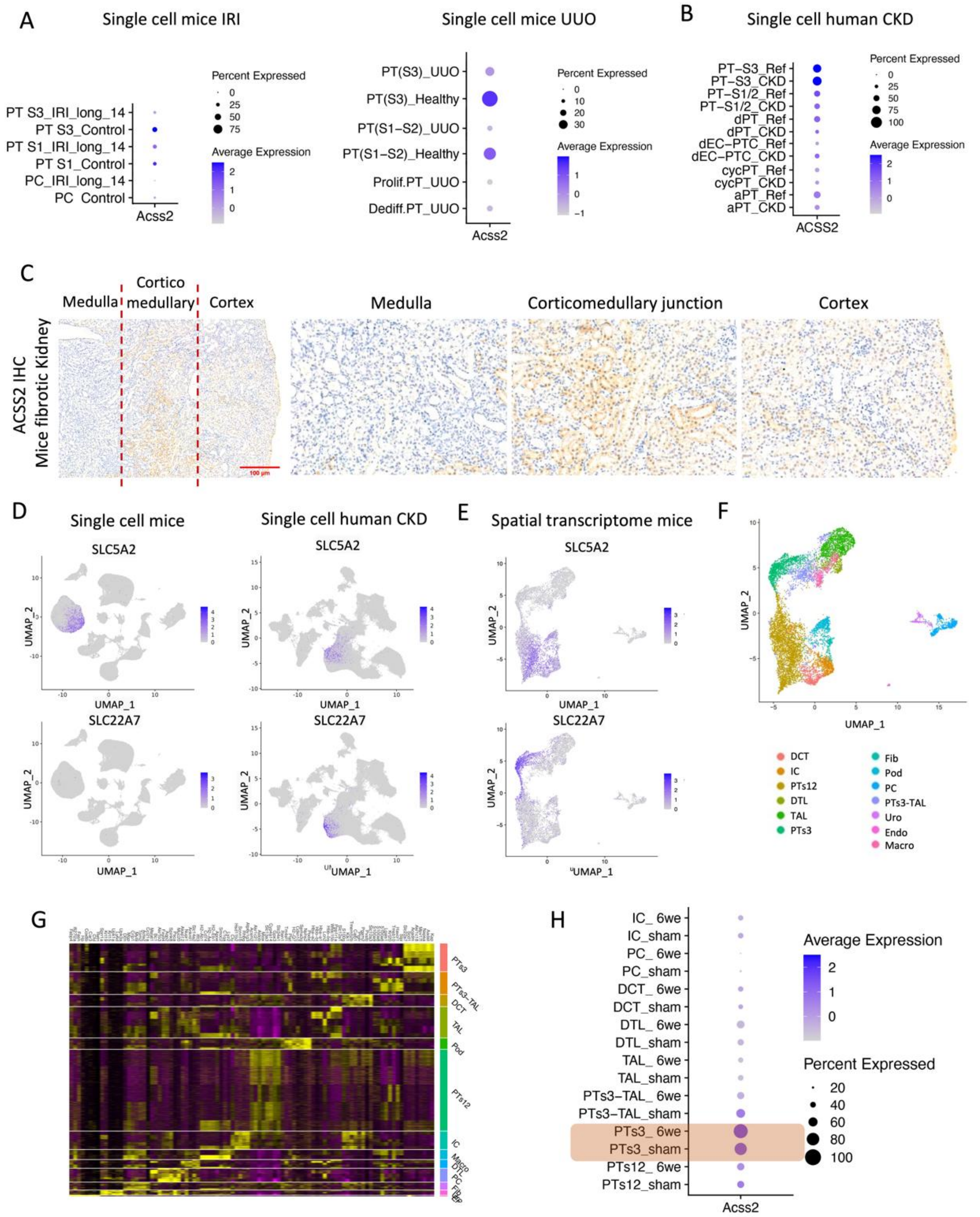
A
Single cell mice IRI
PT S3_IRI_long_14
PT S3_Control
PT S1_IRI_long_14
PT S1_Control
PC_IRI_long_14
PC Control
Acss2
Percent Expressed
Average Expression
Single cell mice UUO
PT(S3)_UUO
PT(S3)_Healthy
PT(S1–S2)_UUO
PT(S1–S2)_Healthy
Prolif.PT_UUO
Dediff.PT_UUO
B
Single cell human CKD
PT–S3_Ref
PT–S3_CKD
PT–S1/2_Ref
PT–S1/2_CKD
dPT_Ref
dPT_CKD
dEC–PTC_Ref
dEC–PTC_CKD
cycPT_Ref
cycPT_CKD
aPT_Ref
aPT_CKD
ACSS2
C
ACSS2 IHC
Mice fibrotic Kidney
Medulla
Cortico medullary
Cortex
Corticomedullary junction
100 µm
D
Single cell mice
Single cell human CKD
SLC5A2
SLC22A7
UMAP_1
UMAP_2
E
Spatial transcriptome mice
F
DCT
IC
PTs12
DTL
TAL
PTs3
Fib
Pod
PC
PTs3-TAL
Uro
Endo
Macro
G
H
IC_ 6we
IC_sham
PC_ 6we
PC_sham
DCT_ 6we
DCT_sham
DTL_ 6we
DTL_sham
TAL_ 6we
TAL_sham
PTs3–TAL_ 6we
PTs3–TAL_sham
PTs3_ 6we
PTs3_sham
PTs12_ 6we
PTs12_sham
Acss2
Average Expression
Percent Expressed

Supplementary Fig. 7. Global genetic knock out of ACSS2 influenced crotonylation and acetylation levels of several histone lysine residues in UUO-induced fibrotic kidneys. (A) The expression of crotonylation levels of H3K18, H3K27, H3K4, H3K23, H3K36 and H3K14 in UUO-induced fibrotic kidneys from wilt type and $ACSS2^{-/-}$mice. (B) The expression of acetylation levels of H3K18, H3K27, H3K4, H3K23, H3K36 and H3K14 in UUO-induced fibrotic kidneys from wilt type and $ACSS2^{-/-}$ mice. (C-D) Protein expression of crotonylation and acetylation levels of several histone lysine residues in whole kidney lysates of control and UUO of WT and $ACSS2^{-/-}$ mice (n = 3 per group). UUO: unilateral ureteric obstruction; WT: wild type; $ACSS2^{-/-}$: ACSS2 knockout mice. Data shown are means ± SEM. Statistical analysis by ANOVA analysis. * Means WT UUO vs. WT Control. # Means WT UUO vs. $ACSS2^{-/-}$ UUO. *$P < 0.05$, **$P < 0.01$, and ***$P < 0.001$. $^{\#}P < 0.05$, and $^{\#\#}P < 0.01$.

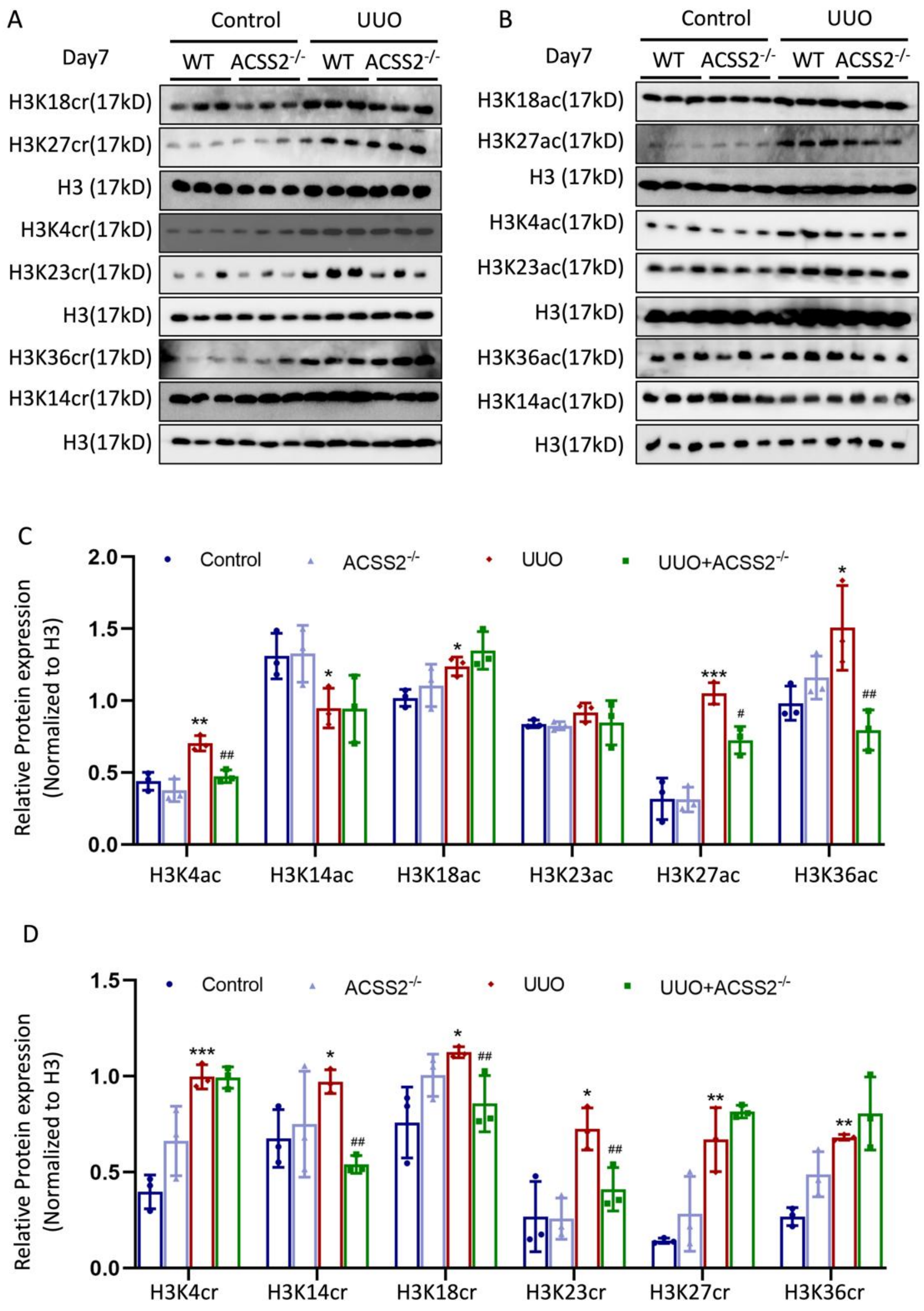

A
Control
UUO
Day7
WT
ACSS2-/-
WT
ACSS2-/-
H3K18cr(17kD)
H3K27cr(17kD)
H3 (17kD)
H3K4cr(17kD)
H3K23cr(17kD)
H3(17kD)
H3K36cr(17kD)
H3K14cr(17kD)
H3(17kD)
B
Control
UUO
Day7
WT
ACSS2-/-
WT
ACSS2-/-
H3K18ac(17kD)
H3K27ac(17kD)
H3 (17kD)
H3K4ac(17kD)
H3K23ac(17kD)
H3(17kD)
H3K36ac(17kD)
H3K14ac(17kD)
H3(17kD)
C
Control
ACSS2-/-
UUO
UUO+ACSS2-/-
Relative Protein expression
(Normalized to H3)
2.0
1.5
1.0
0.5
0.0
H3K4ac
H3K14ac
H3K18ac
H3K23ac
H3K27ac
H3K36ac
D
Control
ACSS2-/-
UUO
UUO+ACSS2-/-
Relative Protein expression
(Normalized to H3)
1.5
1.0
0.5
0.0
H3K4cr
H3K14cr
H3K18cr
H3K23cr
H3K27cr
H3K36cr

Supplementary Fig. 8. Overexpression of ACSS2 in tubular epithelial cells treated with TGFβ1 influenced crotonylation and acetylation levels of several histone lysine residues. (A) The crotonylation levels of H3K14, H3K9, H3K18, H3K4, H3K23, H3K36 and H3K27 in tubular epithelial cells treated with TGFβ with/without ACSS2 plasmids. (B) The acetylation levels of H3K14, H3K9, H3K18, H3K4, H3K23, H3K36 and H3K27 in tubular epithelial cells treated with TGFβ with/without ACSS2 plasmids. (C-D) The crotonylation and acetylation levels of several histone lysine residues in tubular epithelial cells treated with TGFβ with/without ACSS2 plasmids ($n = 3$ per group). ACSS2 OE: ACSS2 overexpression plasmids. Data shown are means ± SEM. Statistical analysis by t-test. *$P < 0.05$, and **$P < 0.01$.

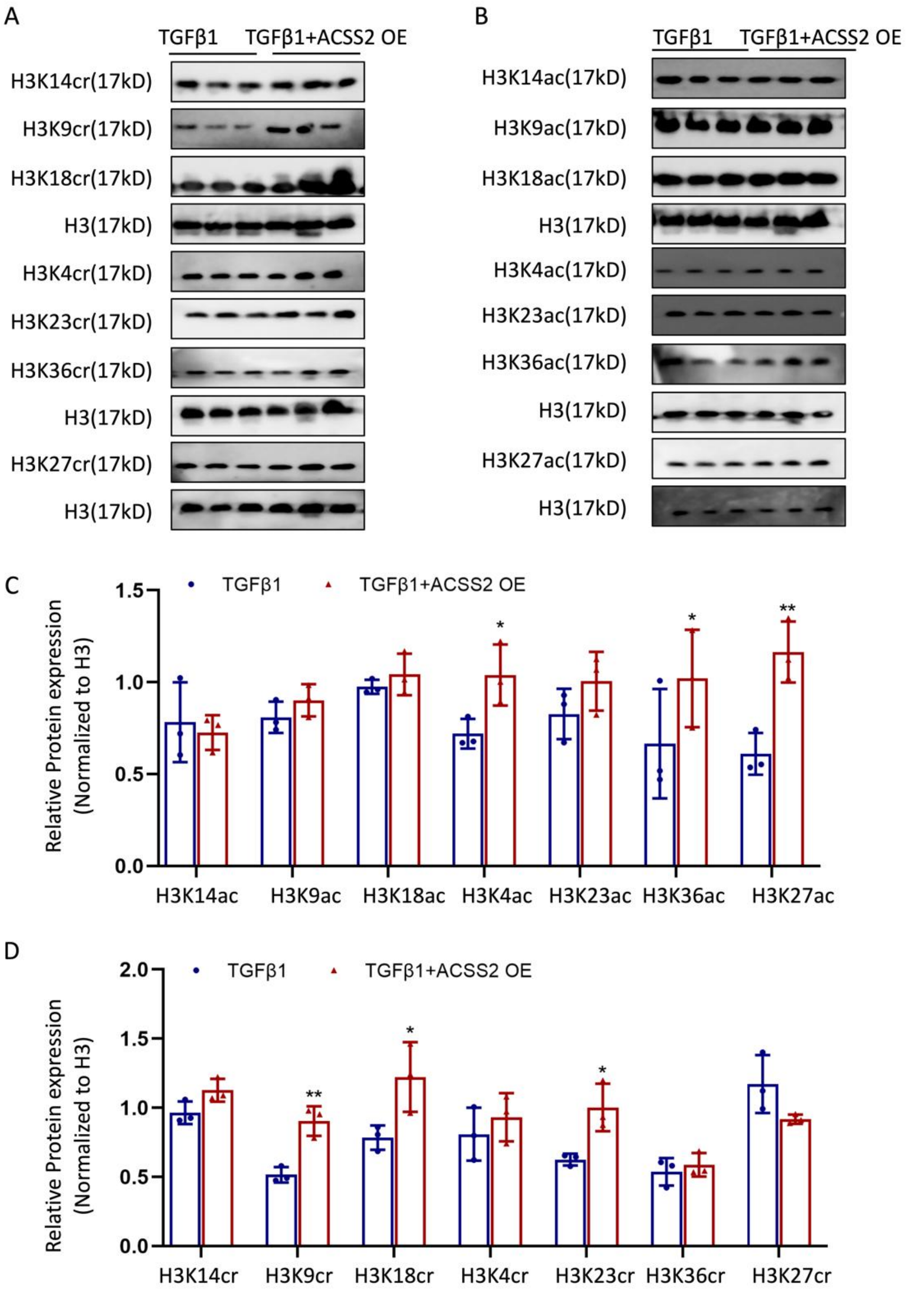

A
TGFβ1
TGFβ1+ACSS2 OE
H3K14cr(17kD)
H3K9cr(17kD)
H3K18cr(17kD)
H3(17kD)
H3K4cr(17kD)
H3K23cr(17kD)
H3K36cr(17kD)
H3(17kD)
H3K27cr(17kD)
H3(17kD)
B
TGFβ1
TGFβ1+ACSS2 OE
H3K14ac(17kD)
H3K9ac(17kD)
H3K18ac(17kD)
H3(17kD)
H3K4ac(17kD)
H3K23ac(17kD)
H3K36ac(17kD)
H3(17kD)
H3K27ac(17kD)
H3(17kD)
C
TGFβ1
TGFβ1+ACSS2 OE
Relative Protein expression (Normalized to H3)
1.5
1.0
0.5
0.0
H3K14ac
H3K9ac
H3K18ac
H3K4ac
H3K23ac
H3K36ac
H3K27ac
D
TGFβ1
TGFβ1+ACSS2 OE
Relative Protein expression (Normalized to H3)
2.0
1.5
1.0
0.5
0.0
H3K14cr
H3K9cr
H3K18cr
H3K4cr
H3K23cr
H3K36cr
H3K27cr

Supplementary Fig. 9. Global genetic knockout of ACSS2 alleviated UUO and folic acid (FA)- induced kidney fibrosis in mice. (A) Protein level of H3K9cr, H3K9ac and H3 in whole kidney lysates of control and FAN of WT and $ACSS2^{-/-}$ mice (n = 3 per group). (B) Quantification of H3K9cr immunoblots normalizing to H3 (n = 3 per group). (C-E) Quantitative analysis of FN1, COL6 and α-SMA in UUO of WT and $ACSS2^{-/-}$ mice (n = 3 per group). (F-G) IF staining and quantitative analysis of α-SMA (green) and DAPI (blue) in UUO of WT and $ACSS2^{-/-}$ mice (n = 3 per group).Scale bar: 100 μm. (H-I) Representative images of H&E and Masson staining in FAN of WT and $ACSS2^{-/-}$ mice. Scale bar: upper panels: 100 μm; lower panels:20 μm. (n= 3 per group). (J) FN1, COL6, α-SMA and GAPDH immunoblotting in the whole kidney lysates of control and FAN of WT and $ACSS2^{-/-}$ mice (n = 3 per group). (K) The mRNA levels of Fn1, Col1a1 and Acta2 in whole kidney lysates of WT and $ACSS2^{-/-}$ mice injected with FA (n = 3 to 5 per group). FAN: folic acid nephropathy; UUO: unilateral ureteric obstruction; WT: wild type; $ACSS2^{-/-}$: ACSS2 knockout; IF: Immunofluorescence; Data shown are means ± SEM. Statistical analysis by one-way ANOVA with Tukey's post hoc test. *$P < 0.05$, ***$P < 0.001$ and ****$P < 0.0001$.

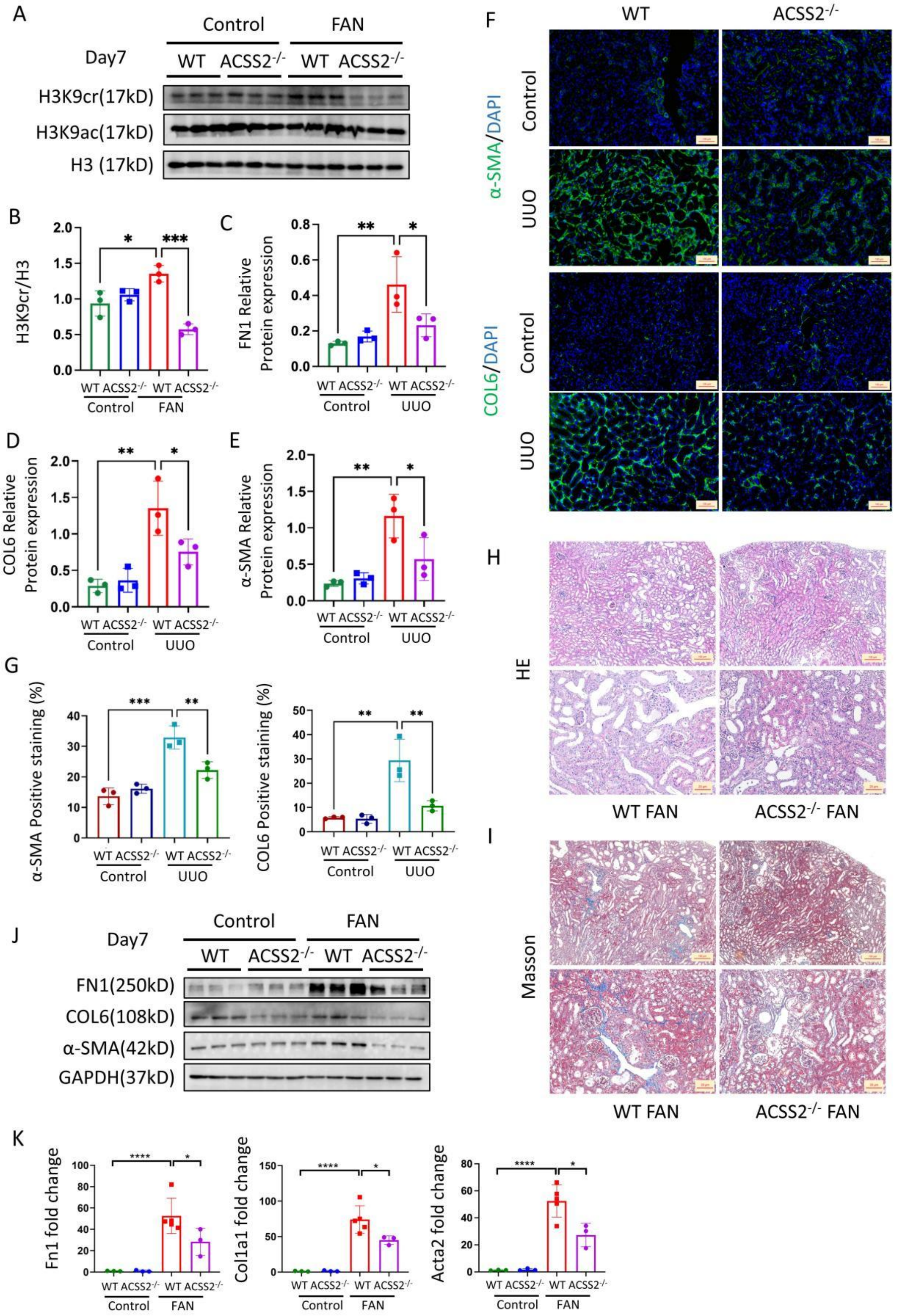

A
Control
FAN
Day7
WT ACSS2-/- WT ACSS2-/-
H3K9cr(17kD)
H3K9ac(17kD)
H3 (17kD)
B
H3K9cr/H3
C
FN1 Relative Protein expression
D
COL6 Relative Protein expression
E
α-SMA Relative Protein expression
F
WT
ACSS2-/-
α-SMA/DAPI
COL6/DAPI
Control
UUO
G
α-SMA Positive staining (%)
COL6 Positive staining (%)
H
HE
WT FAN
ACSS2-/- FAN
I
Masson
J
Day7
FN1(250kD)
COL6(108kD)
α-SMA(42kD)
GAPDH(37kD)
K
Fn1 fold change
Col1a1 fold change
Acta2 fold change

Supplementary Fig. 10. Generation of renal tubular epithelial cell (TEC)-specific ACSS2 knockout mice. (A) Representative IHC staining and quantitative analysis of ACSS2 in healthy control subjects and patients with chronic kidney diseases. Scale bar: 50 μm. (n= 3 per group). (B) Schematic of ACSS2$^{flox/flox}$ (ACSS2$^{f/f}$) mice generation by CRISPR/Cas9-stimulated homologous recombination. (C-D) Identification of the genotype of ACSS2$^{f/f}$ mice and ACSS2^{tecKO} (Cdh16-Cre+ ACSS2$^{f/f}$) mice by PCR assay. IHC: immunohistochemical; FSGS: fuel sales grant scheme; ANCA: anti-neutrophil cytoplasmic antibodies; LN: lupus nephritis. Statistical analysis by Pearson correlation.

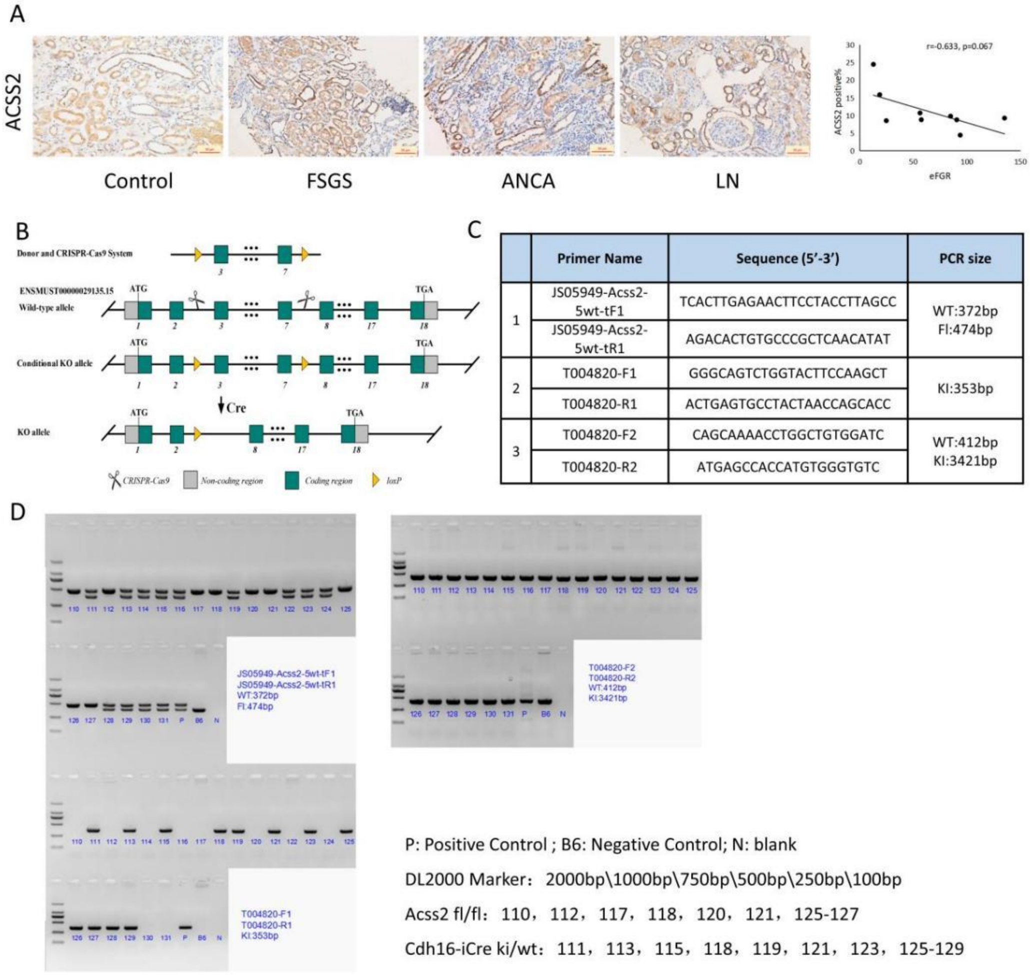

Supplementary Fig. 11. TEC-specific deletion of ACSS2 suppressed H3K9cr level and alleviated UUO and FA-induced kidney fibrosis in mice. (A) H3K9cr, H3K9ac and H3 immunoblotting and quantification of these immunoblots in the whole kidney lysates of control and FAN of WT and $ACSS2^{tecKO}$ mice (n = 3 per group). (B) Quantification of FN1, COL1a1, COL6 and GAPDH immunoblotting in the whole kidney lysates of control and UUO of WT and $ACSS2^{tecKO}$ mice (n = 3 per group). (C) mRNA levels of Col1a1 or Col6 in whole kidney lysates of WT and $ACSS2^{tecKO}$ mice treated with FAN (n = 6 per group). (D) COL1a1, COL6 and GAPDH immunoblotting and quantification of these immunoblots in the whole kidney lysates of control and FAN of WT and $ACSS2^{tecKO}$ mice (n = 3 per group). FAN: folic acid nephropathy; UUO: unilateral ureteric obstruction; WT: wild type; ACSS2 CKO: tubular epithelial cell-specific knockout of ACSS2. Data shown are means ± SEM. Statistical analysis by one-way ANOVA with Tukey's post hoc test. $^{**}P < 0.01$, $^{***}P < 0.001$ and $^{****}P < 0.0001$.

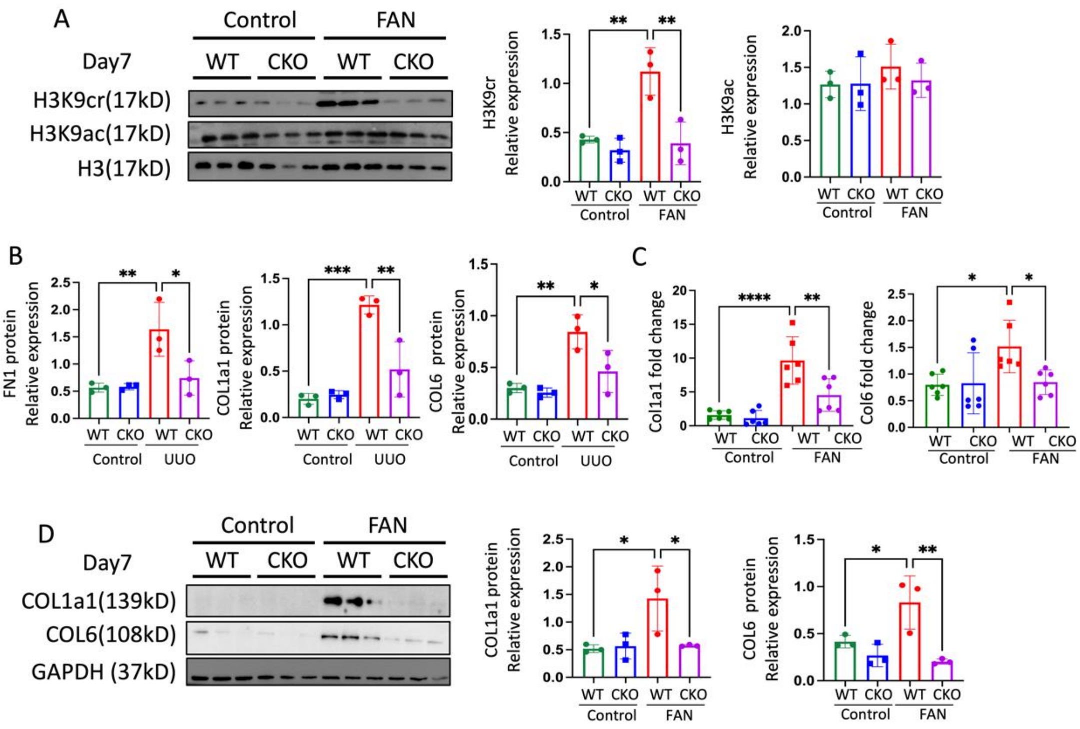
A
Control
FAN
Day7
WT
CKO
WT
CKO
H3K9cr(17kD)
H3K9ac(17kD)
H3(17kD)
H3K9cr
Relative expression
H3K9ac
Relative expression
WT CKO WT CKO
Control FAN
B
FN1 protein
Relative expression
COL1a1 protein
Relative expression
COL6 protein
Relative expression
WT CKO WT CKO
Control UUO
C
Col1a1 fold change
Col6 fold change
WT CKO WT CKO
Control FAN
D
Control
FAN
Day7
WT
CKO
WT
CKO
COL1a1(139kD)
COL6(108kD)
GAPDH (37kD)
COL1a1 protein
Relative expression
COL6 protein
Relative expression
WT CKO WT CKO
Control FAN

Supplementary Fig. 12. The ChIP-seq analysis in each sample. (A) ChIP-seq analysis of test chromatin occupancy and heatmap of the distribution around±3 kb from the TSSs of H3K9ac in WT control mice. **(**B) ChIP-seq analysis of test chromatin occupancy and heatmap of the distribution around±3 kb from the TSSs of H3K9cr in WT control mice. **(**C) ChIP-seq analysis of test chromatin occupancy and heatmap of the distribution around±3 kb from the TSSs of input in WT control mice. **(**D) ChIP-seq analysis of test chromatin occupancy and heatmap of the distribution around±3 kb from the TSSs of H3K9ac in ACSS2$^{-/-}$ control mice. **(**E) ChIP-seq analysis of test chromatin occupancy and heatmap of the distribution around±3 kb from the TSSs of H3K9cr in ACSS2$^{-/-}$ control mice. **(**F) ChIP-seq analysis of test chromatin occupancy and heatmap of the distribution around±3 kb from the TSSs of input in ACSS2$^{-/-}$ control mice. **(**G) ChIP-seq analysis of test chromatin occupancy and heatmap of the distribution around±3 kb from the TSSs of H3K9ac in WT UUO mice. **(**H) ChIP-seq analysis of test chromatin occupancy and heatmap of the distribution around±3 kb from the TSSs of H3K9cr in WT UUO mice. **(**I) ChIP-seq analysis of test chromatin occupancy and heatmap of the distribution around±3 kb from the TSSs of input in WT UUO mice. **(**J) ChIP-seq analysis of test chromatin occupancy and heatmap of the distribution around±3 kb from the TSSs of H3K9ac in ACSS2$^{-/-}$ UUO mice. **(**K) ChIP-seq analysis of test chromatin occupancy and heatmap of the distribution around±3 kb from the TSSs of H3K9cr in ACSS2$^{-/-}$ UUO mice. **(**L) ChIP-seq analysis of test chromatin occupancy and heatmap of the distribution

around±3 kb from the TSSs of input in ACSS2$^{-/-}$ UUO mice. UUO: unilateral ureteric obstruction; WT: wild type; ACSS2$^{-/-}$: ACSS2 knockout.

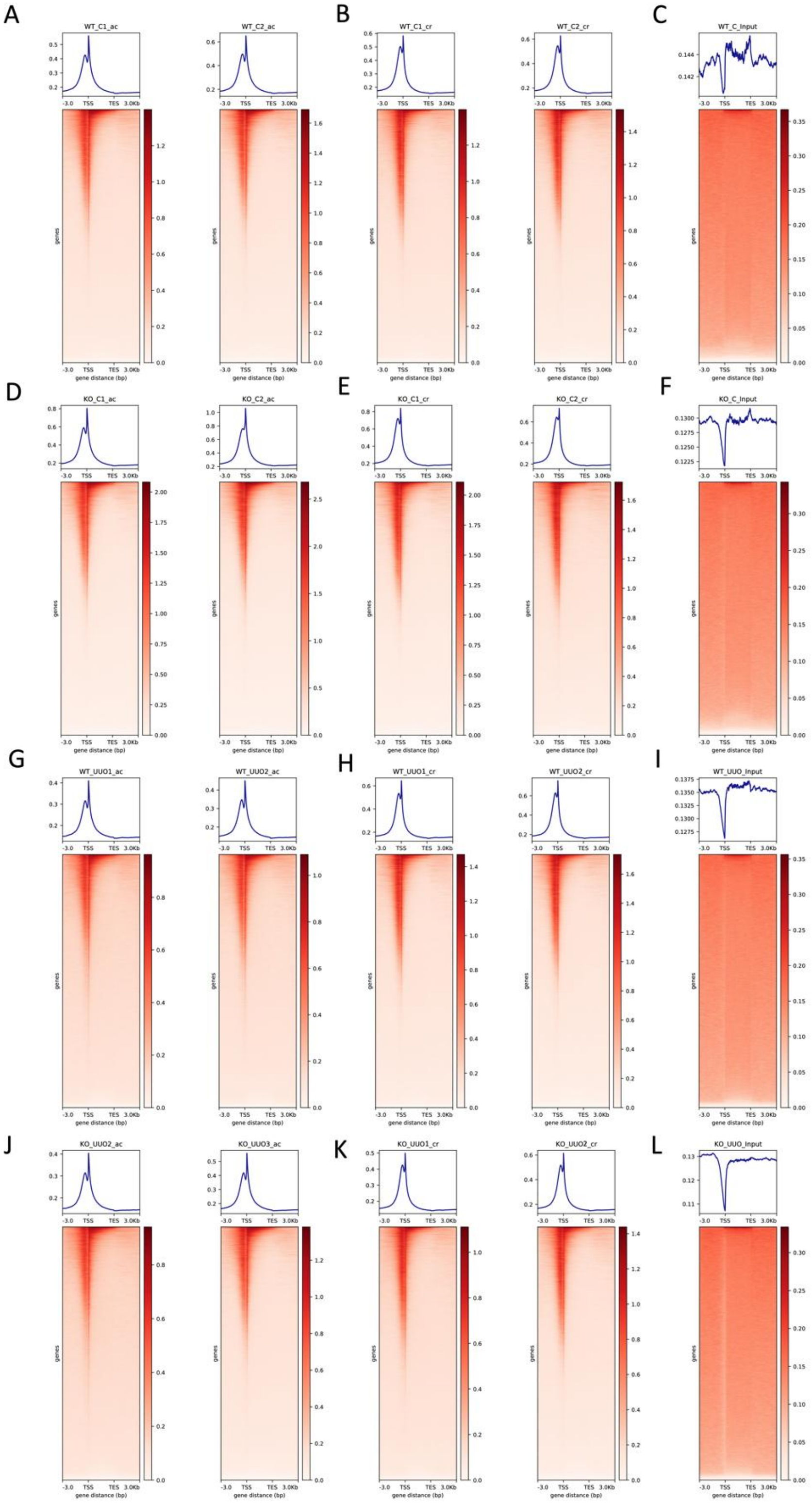

Supplementary Fig. 13. The combination analysis of ChIP sequencing and RNA sequencing. (A) H3K9cr and H3K9ac ChIP signals are shown in both control and UUO mice. (B) Mean H3K9cr to H3K9ac ChIP ratios in the control of WT and ACSS2$^{-/-}$ mice. (C-D) Comparable analysis from RNA sequencing between control and UUO group using KEGG and GO database (Fc>2, P-adj<0.05). (E-F) Comparable analysis from RNA sequence between UUO of WT and ACSS2$^{-/-}$ group using KEGG and GO database (Fc>2, P-adj<0.05). (G) Enrichment plots from the GSEA results, including 'cytokine-cytokine receptor interaction', and 'response to interleukin-1'. "Gene count" is the number of genes enriched in a GO (gene ontology) or KEGG (Kyoto Encyclopedia of Genes and Genomes) term. 'Gene ratio' is the percentage of total differential expression genes in the given GO/KEGG term. UUO: unilateral ureteric obstruction; WT: wild type; ACSS2$^{-/-}$: ACSS2 knockout; WT_C_ac: H3K9ac ChIP Signals in control mice; WT_UUO_ac: H3K9ac ChIP Signals in UUO mice; WT_C_cr: H3K9cr ChIP Signals in control mice; WT_UUO_cr: H3K9cr ChIP Signals in UUO mice.

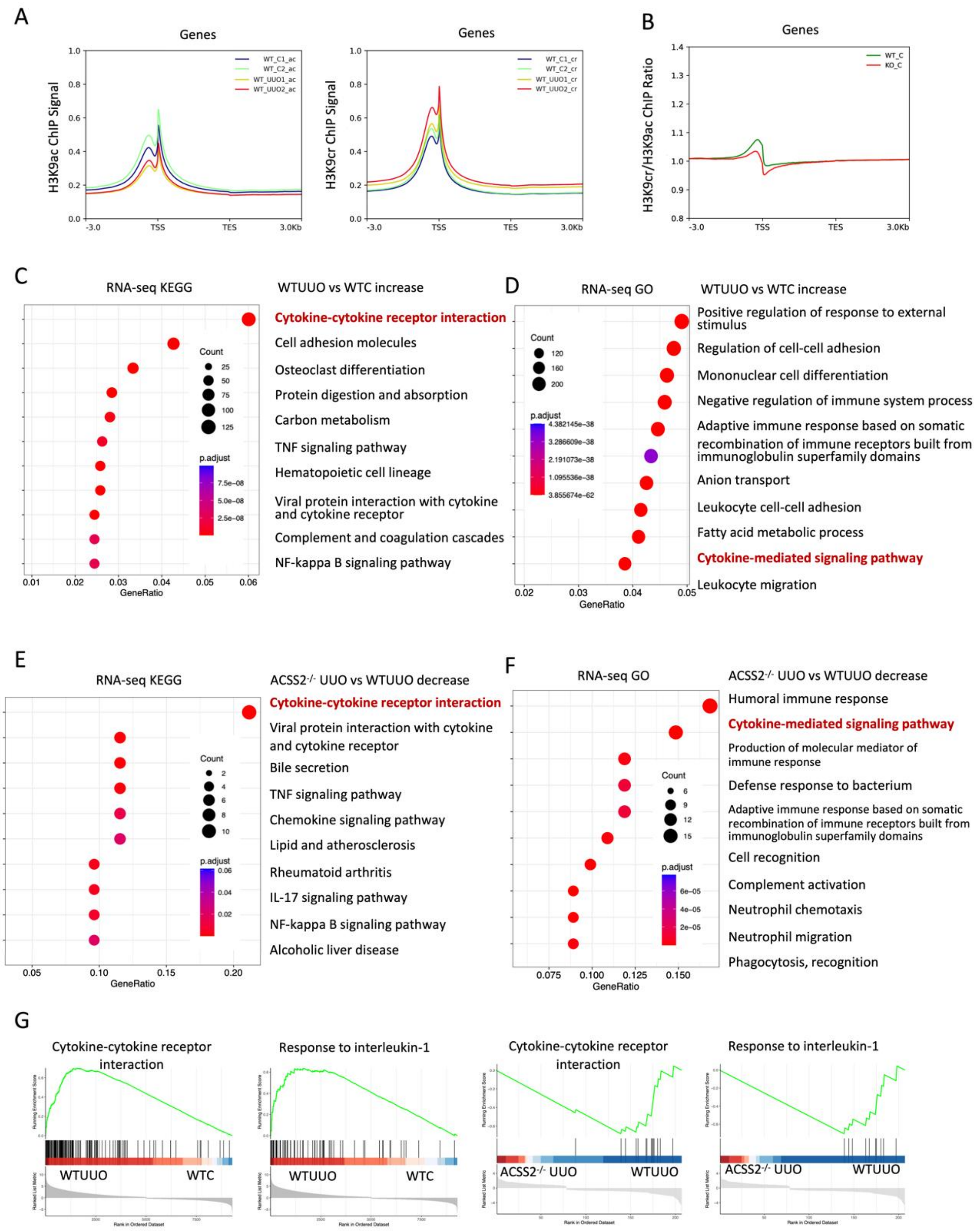
A
Genes
H3K9ac ChIP Signal
WT_C1_ac
WT_C2_ac
WT_UUO1_ac
WT_UUO2_ac
-3.0
TSS
TES
3.0Kb
Genes
H3K9cr ChIP Signal
WT_C1_cr
WT_C2_cr
WT_UUO1_cr
WT_UUO2_cr
B
Genes
H3K9cr/H3K9ac ChIP Ratio
WT_C
KO_C
C
RNA-seq KEGG
WTUUO vs WTC increase
Cytokine-cytokine receptor interaction
Cell adhesion molecules
Osteoclast differentiation
Protein digestion and absorption
Carbon metabolism
TNF signaling pathway
Hematopoietic cell lineage
Viral protein interaction with cytokine and cytokine receptor
Complement and coagulation cascades
NF-kappa B signaling pathway
Count
p.adjust
GeneRatio
D
RNA-seq GO
WTUUO vs WTC increase
Positive regulation of response to external stimulus
Regulation of cell-cell adhesion
Mononuclear cell differentiation
Negative regulation of immune system process
Adaptive immune response based on somatic recombination of immune receptors built from immunoglobulin superfamily domains
Anion transport
Leukocyte cell-cell adhesion
Fatty acid metabolic process
Cytokine-mediated signaling pathway
Leukocyte migration
Count
p.adjust
GeneRatio
E
RNA-seq KEGG
ACSS2-/- UUO vs WTUUO decrease
Cytokine-cytokine receptor interaction
Viral protein interaction with cytokine and cytokine receptor
Bile secretion
TNF signaling pathway
Chemokine signaling pathway
Lipid and atherosclerosis
Rheumatoid arthritis
IL-17 signaling pathway
NF-kappa B signaling pathway
Alcoholic liver disease
Count
p.adjust
GeneRatio
F
RNA-seq GO
ACSS2-/- UUO vs WTUUO decrease
Humoral immune response
Cytokine-mediated signaling pathway
Production of molecular mediator of immune response
Defense response to bacterium
Adaptive immune response based on somatic recombination of immune receptors built from immunoglobulin superfamily domains
Cell recognition
Complement activation
Neutrophil chemotaxis
Neutrophil migration
Phagocytosis, recognition
Count
p.adjust
GeneRatio
G
Cytokine-cytokine receptor interaction
Response to interleukin-1
Cytokine-cytokine receptor interaction
Response to interleukin-1
WTUUO
WTC
WTUUO
WTC
ACSS2-/- UUO
WTUUO
ACSS2-/- UUO
WTUUO

Supplementary Fig. 14. The combination analysis of ChIP sequencing/RNA sequencing data and the specificity of transcription factor binding motifs associated with H3K9cr.(A) Genome browser representation of RNA-seq reads and ChIP-seq reads for Il1r1 from UUO of control and ACSS2$^{-/-}$ mice. (B-C) qPCR analysis of H3K9Cr, H3K4cr, H3K14cr, H3K18cr, H3K23cr, H3K27cr, H3K36cr and H2BK34cr ChIP products from HEK-293T cells transfected with ACSS2 plasmids for 24 hours. (D) DNA motifs enriched of H3k9cr in WT and UUO determined by HOMER motif analysis. UUO: unilateral ureteric obstruction; WT: wild type; ACSS2$^{-/-}$: ACSS2 knockout. Data are represented as mean of fold enrichment ± standard deviation of technical replicates.

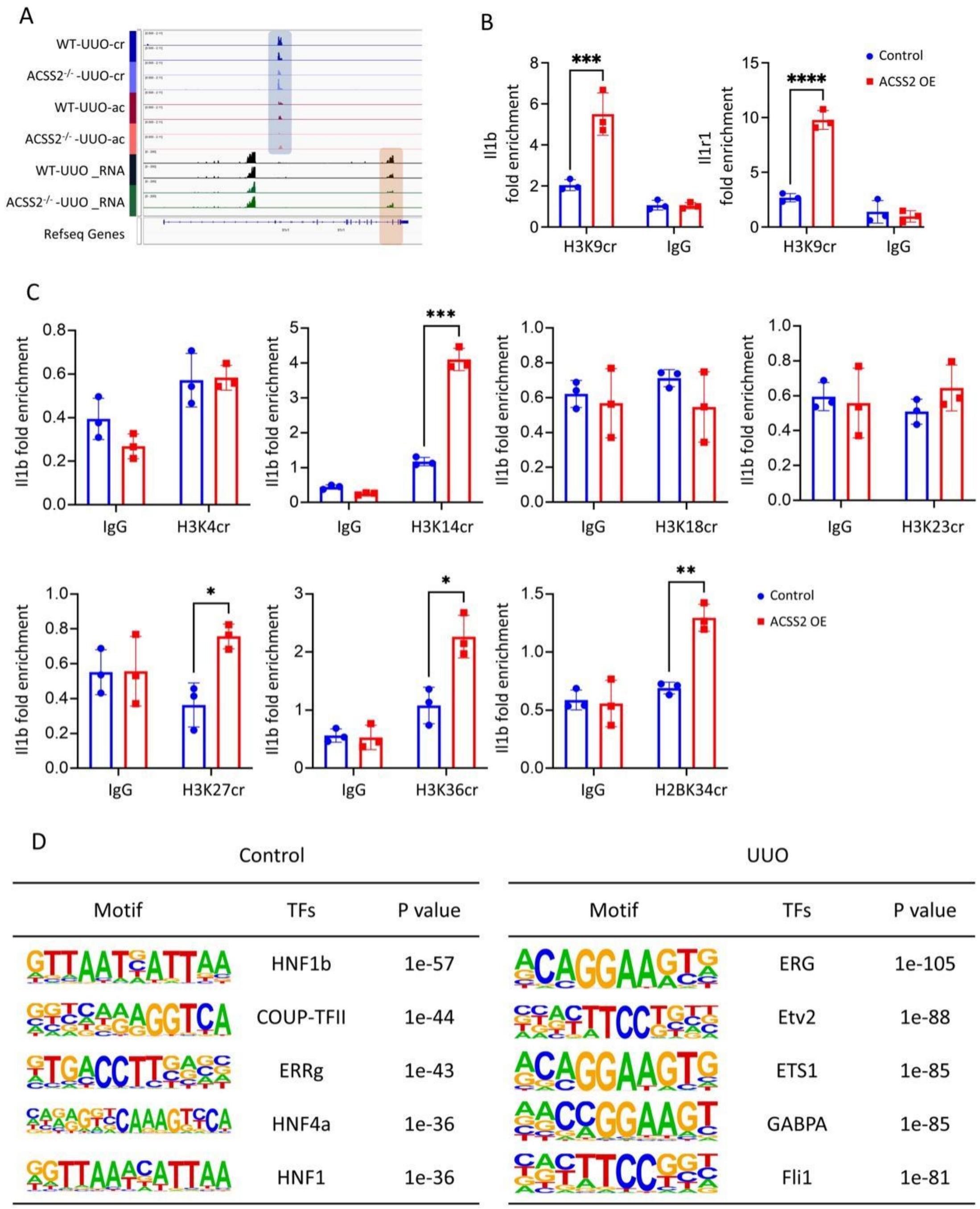
A
WT-UUO-cr
ACSS2-/- -UUO-cr
WT-UUO-ac
ACSS2-/- -UUO-ac
WT-UUO _RNA
ACSS2-/- -UUO _RNA
Refseq Genes
B
Il1b fold enrichment
H3K9cr
IgG
***
Il1r1 fold enrichment
****
Control
ACSS2 OE
C
Il1b fold enrichment
IgG
H3K4cr
H3K14cr
H3K18cr
H3K23cr
H3K27cr
H3K36cr
H2BK34cr
***
*
*
**
Control
ACSS2 OE
D
Control
Motif | TFs | P value
HNF1b | 1e-57
COUP-TFII | 1e-44
ERRg | 1e-43
HNF4a | 1e-36
HNF1 | 1e-36
UUO
Motif | TFs | P value
ERG | 1e-105
Etv2 | 1e-88
ETS1 | 1e-85
GABPA | 1e-85
Fli1 | 1e-81

Supplementary Fig. 15. IL-1β expression and the correlation between IL-1β with ACSS2 and H3K9cr. (A) Representative IHC staining with IL-1β antibody in healthy control subjects and patients with chronic kidney diseases. Scale bar: upper panels: 50 μm; lower panels:20 μm.(n= 3 per group). (B) Quantitative IHC analysis of IL-1β expression in control and patients with chronic kidney diseases using ImageJ 6.0 software. (C) Expression of ACSS2, IL-1β, IL1r1 and IL1rap for tubular epithelial cells in mice from spatial transcription analysis. Cell types include distal convoluted tubules (DCT), descending limb (DTL), ascending limb (TAL), proximal tubule segments 1-2 (PTs12), proximal tubule segment 3 (PTs3), and proximal tubule segment 3-thick ascending limb (PTs3-TAL). (D) IF staining of H3k9cr (yellow), IL-1β (red) and DAPI (blue) in UUO of WT and $ACSS2^{-/-}$ mice. Scale bar: 25 μm. (n= 2 per group). WT: wild type; UUO: unilateral ureteric obstruction; $ACSS2^{-/-}$: ACSS2 knockout; FSGS: fuel sales grant scheme; ANCA: anti-neutrophil cytoplasmic antibodies; MN: membranous nephropathy. IHC: immunohistochemical; IF: immunofluorescence; Sham: Sham surgery mice; 6we: 6 weeks after ischemia reperfusion injury. Statistical analysis by Pearson correlation.

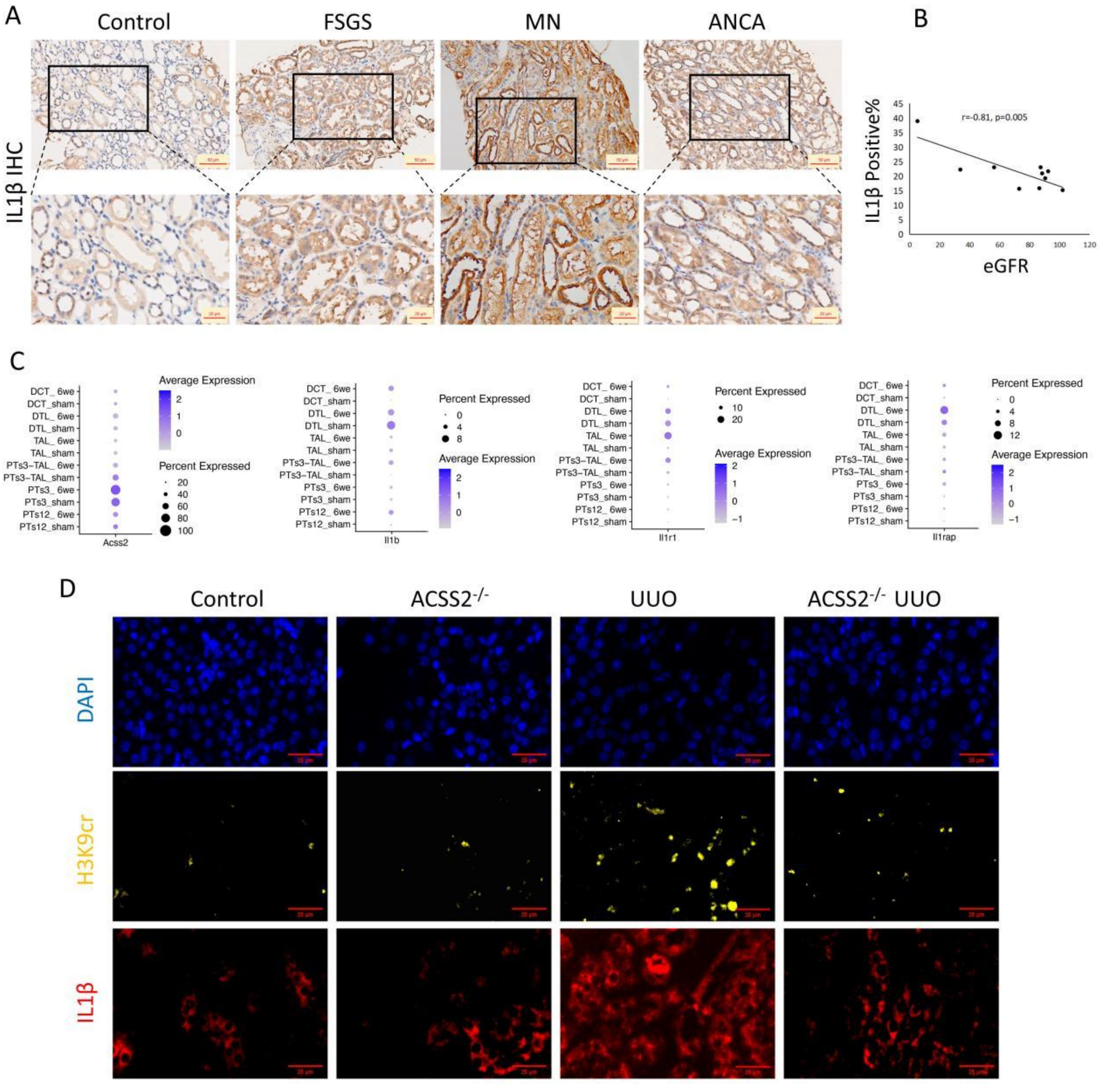
A
Control
FSGS
MN
ANCA
IL1β IHC
B
r=-0.81, p=0.005
IL1β Positive%
eGFR
C
Average Expression
Percent Expressed
Acss2
Il1b
Il1r1
Il1rap
D
Control
ACSS2-/-
UUO
ACSS2-/- UUO
DAPI
H3K9cr
IL1β

Supplementary Fig. 16. The changes of IL-1β expression in kidneys. (A) Immunoblotting and quantification of IL-1β immunoblots normalizing to GAPDH in the whole kidney lysates of control and mice treated with UUO or injected with folic acid (n = 5 to 6 per group). (B) The mRNA levels of IL-1β in the whole kidney lysates of control and mice treated with UUO or injected with folic acid (n = 3 to 6 per group). (C) Immunoblotting and quantification of IL-1β immunoblots normalizing to GAPDH as well as IL-1β mRNA levels in control and FAN of WT and ACSS2$^{-/-}$ mice (n = 3 to 6 per group). (D) Immunoblotting and quantification of IL-1β immunoblots normalizing to GAPDH as well as IL-1β mRNA levels in control and FAN of WT and ACSS2^{tecKO} mice (n = 3 to 6 per group). Ctrl: control; WT: wild type; UUO: unilateral ureteric obstruction; ACSS2$^{-/-}$: ACSS2 knockout. ACSS2 CKO: TEC-specific deletion of ACSS2. Data shown are means ± SEM. Statistical analysis by one-way ANOVA with Tukey's post hoc test. *P < 0.05, **P < 0.01 and ***P < 0.001.

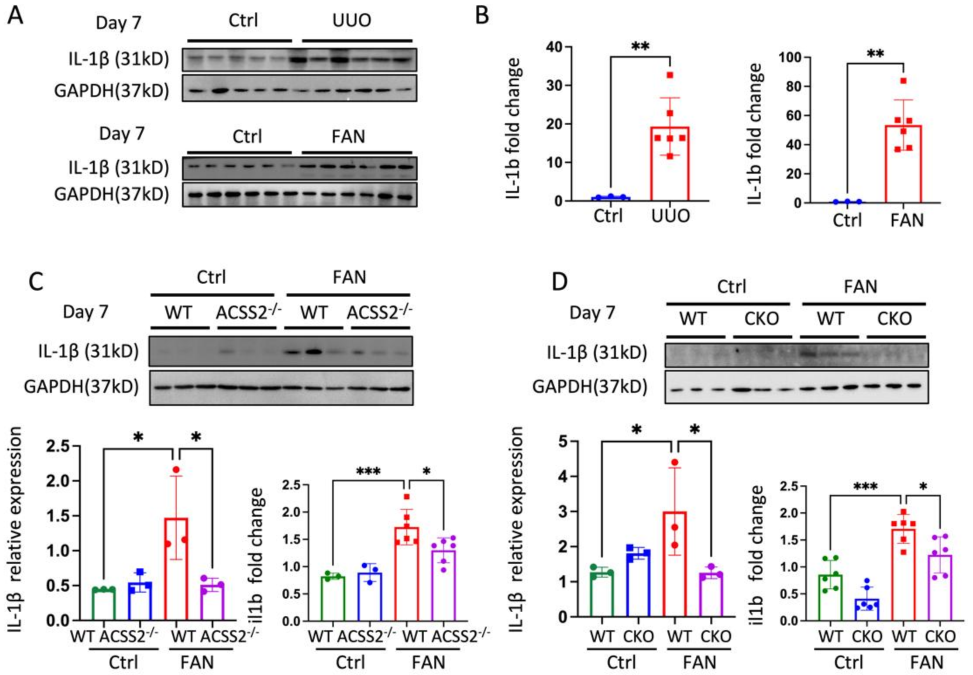

A
Day 7
Ctrl
UUO
IL-1β (31kD)
GAPDH(37kD)
Day 7
Ctrl
FAN
IL-1β (31kD)
GAPDH(37kD)
B
IL-1b fold change
Ctrl
UUO
**
IL-1b fold change
Ctrl
FAN
**
C
Day 7
Ctrl
FAN
WT
ACSS2-/-
IL-1β (31kD)
GAPDH(37kD)
IL-1β relative expression
il1b fold change
D
Day 7
Ctrl
FAN
WT
CKO
IL-1β (31kD)
GAPDH(37kD)
IL-1β relative expression
il1b fold change

Supplementary Fig. 17. The increased IL-1β expression when treated with ACSS2 plasmids or crotonate *in vitro*. (A) Immunoblotting and quantification of IL-1β immunoblots normalizing to GAPDH in TCMK-1 cells treated with 10uM crotonate (n = 3 per group). (B) mRNA levels of IL-1β in TCMK-1 cells treated with 10 μM crotonate (n = 3 per group). (C) Immunoblotting and quantification of IL-1β immunoblots normalizing to GAPDH in HEK-293T cells treated with 10 μM crotonate (n = 3 per group). (D) The mRNA levels of IL-1β in HEK-293T cells treated with 10 μM crotonate (n = 3 per group). (E) IL-1β, cleaved IL-1β and GAPDH immunoblotting in TCMK-1 cells transfected with ACSS2 plasmids (n = 3 per group). (F) IL-1β mRNA levels of TCMK-1 cells transfected with ACSS2 plasmids (n = 6 per group). (G) Cellular supernatants levels of IL-1β in TCMK-1 cells transfected with ACSS2 plasmids by ELISA kit (n = 6 to 8 per group). (H) IL-1β, cleaved IL-1β and GAPDH immunoblotting in HEK-293T cells transfected with ACSS2 plasmids (n = 3 per group). (I) IL-1β mRNA levels of HEK-293T cells transfected with ACSS2 plasmids (n = 6 per group). (J) Cellular supernatants levels of IL-1β in HEK-293T cells transfected with ACSS2 plasmids by ELISA kit (n = 5 per group). (K) ACSS2 mRNA levels of PTC tread with TGFβ (n = 6 per group). (L) Protein expression of ACSS2 and H3K9cr in PTC from WT and $ACSS2^{-/-}$ mice tread with TGFβ were determined by western blotting (n =3 per group). (M) IL-1β mRNA levels of PTC tread with TGFβ (n = 6 per group). Ctrl: Control; CR10: 10μM crotonate; ACSS2 OE: ACSS2 overexpression; PTC: primary tubular epithelial cells. Data shown are means ± SEM. Statistical analysis by t-test. $*P < 0.05$, $**P < 0.01$ and $***P < 0.001$.

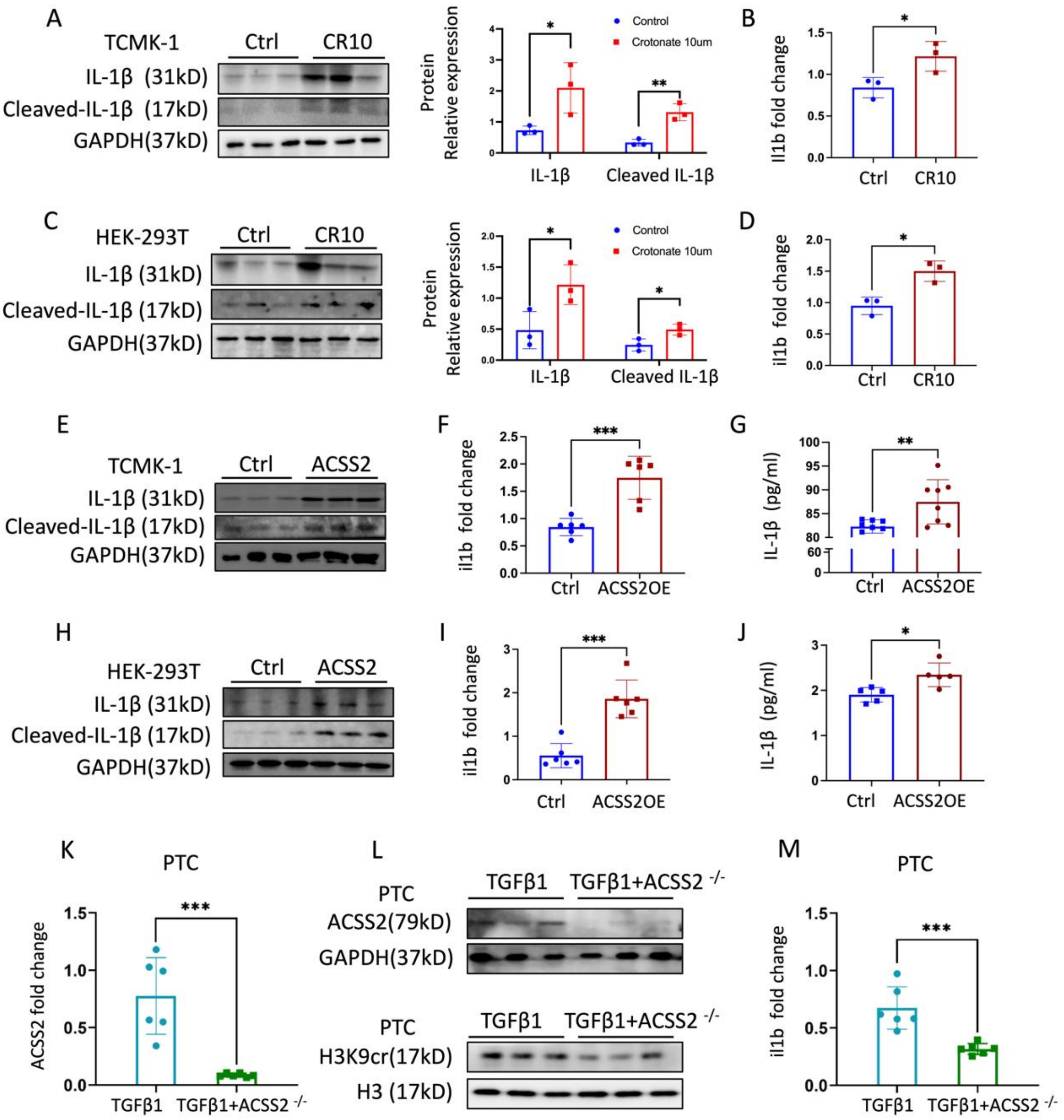

A
TCMK-1
Ctrl
CR10
IL-1β (31kD)
Cleaved-IL-1β (17kD)
GAPDH(37kD)
Protein Relative expression
Control
Crotonate 10um
IL-1β
Cleaved IL-1β
B
Il1b fold change
Ctrl
CR10
C
HEK-293T
Ctrl
CR10
IL-1β (31kD)
Cleaved-IL-1β (17kD)
GAPDH(37kD)
Protein Relative expression
Control
Crotonate 10um
IL-1β
Cleaved IL-1β
D
il1b fold change
Ctrl
CR10
E
TCMK-1
Ctrl
ACSS2
IL-1β (31kD)
Cleaved-IL-1β (17kD)
GAPDH(37kD)
F
il1b fold change
Ctrl
ACSS2OE
G
IL-1β (pg/ml)
Ctrl
ACSS2OE
H
HEK-293T
Ctrl
ACSS2
IL-1β (31kD)
Cleaved-IL-1β (17kD)
GAPDH(37kD)
I
il1b fold change
Ctrl
ACSS2OE
J
IL-1β (pg/ml)
Ctrl
ACSS2OE
K
PTC
ACSS2 fold change
TGFβ1
TGFβ1+ACSS2 -/-
L
PTC
TGFβ1
TGFβ1+ACSS2 -/-
ACSS2(79kD)
GAPDH(37kD)
PTC
TGFβ1
TGFβ1+ACSS2 -/-
H3K9cr(17kD)
H3 (17kD)
M
PTC
il1b fold change
TGFβ1
TGFβ1+ACSS2 -/-

Supplementary Fig. 18. The decreased IL-1β expression when treated with SIRT1/2/3 and HDAC1/2/3 plasmids *in vitro*. (A) IL-1β and GAPDH immunoblotting and quantification of immunoblots normalizing to GAPDH in TCMK-1 and HEK-293T cells transfected with SIRT1/2/3 and HDAC1/2/3 plasmids (n = 3 per group). (B) IL-1β and GAPDH immunoblotting and quantification of immunoblots normalizing to GAPDH in TCMK-1 and HEK-293T cells transfected with SIRT1/2/3 and HDAC1/2/3 plasmids (n = 3 per group). Data shown are means ± SEM. Statistical analysis by one-way ANOVA with Tukey's post hoc test. $^{*}P < 0.05$, $^{**}P < 0.01$ and $^{***}P < 0.001$.

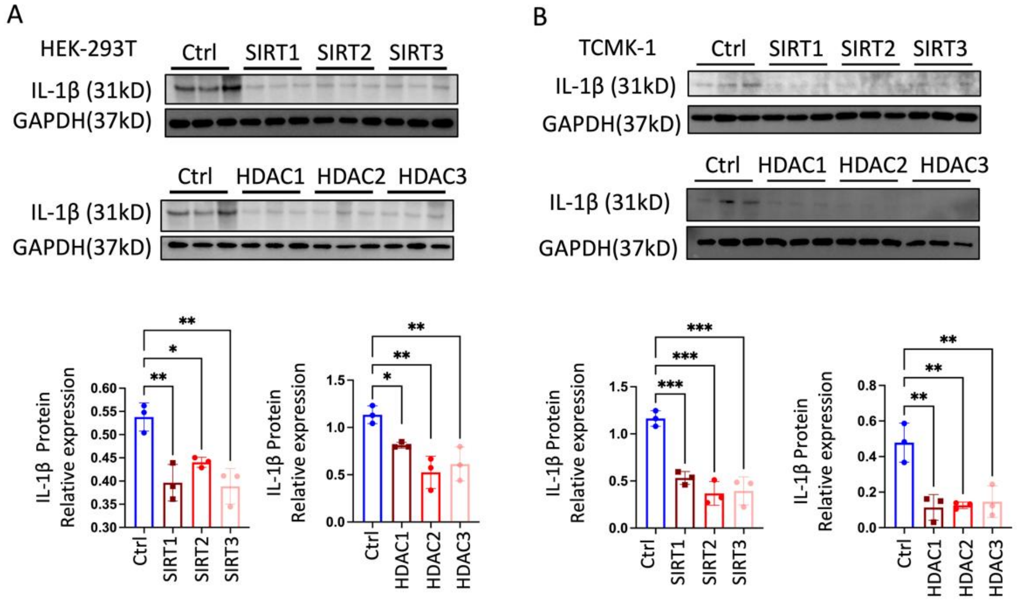

Supplementary Fig. 19. The unchanged IL-1β expression when treated with SIRT4/5/6 plasmids in vitro. (A) IL-1β, cleaved IL-1β and GAPDH immunoblotting and quantification of immunoblots normalizing to GAPDH in TCMK-1 cells transfected with SIRT4/5/6 plasmids (n = 3 per group). (B) IL-1β, cleaved IL-1β and GAPDH immunoblotting and quantification of immunoblots normalizing to GAPDH in HEK-293T cells transfected with SIRT4/5/6 plasmids (n = 3 per group). Data shown are means ± SEM. Statistical analysis by one-way ANOVA with Tukey's post hoc test.

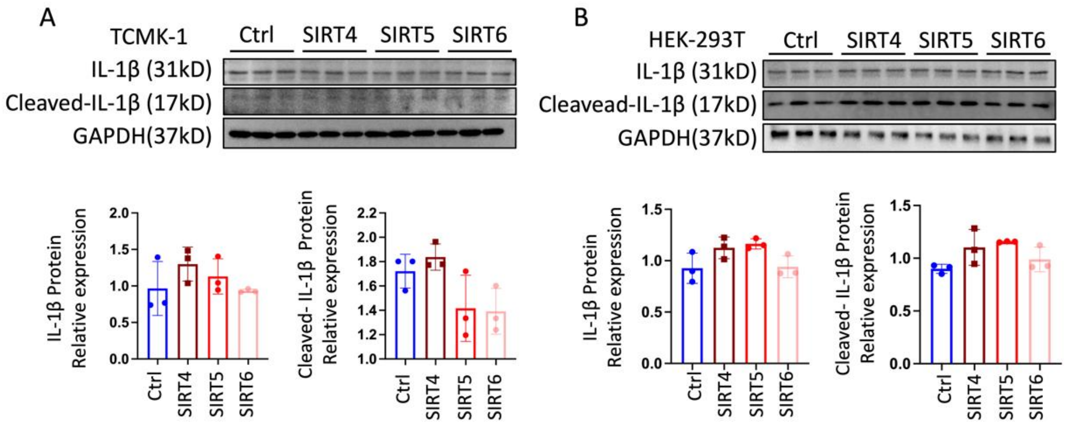

Supplementary Fig. 20. The changes of M1 macrophage polarization when treating IL-1β *in vitro*. (A) Cell type clustering and (B) the expression of ACSS2 and CD68 in kidneys in spatial transcription analysis. Cell types include proximal tubule segments 1–2 (PTs12), proximal tubule segment 3 (PTs3), distal convoluted tubules (DCT), intercalated cells (IC), podocytes (Pod), fibroblasts (Fib), thick ascending limb (TAL), principal cells (PC), urothelium (Uro), and proximal tubule segment 3-thick ascending limb (PTs3-TAL), macrophage (Macro), adipocytes (Adipo). (C) CCK8 results of RAW264.7 stimulating with different doses of IL-1β (n = 6 per group). (D) Microscopic images depicted the changes of RAW264.7 morphology from rounded M0 phenotype to flat M1 phenotype after IL-1β simulation. Scale bar: upper panels: 50 μm; lower panels:20 μm. (E) Tnf-α, iNOS, IL-1β and Cd206 mRNA levels of RAW264.7 which was stimulated with IL-1β under different doses (n = 5 per group). Data shown are means ± SEM. Statistical analysis by one-way ANOVA with Tukey's post hoc test. *P < 0.05, **P < 0.01, ***P < 0.001 and ****P < 0.0001

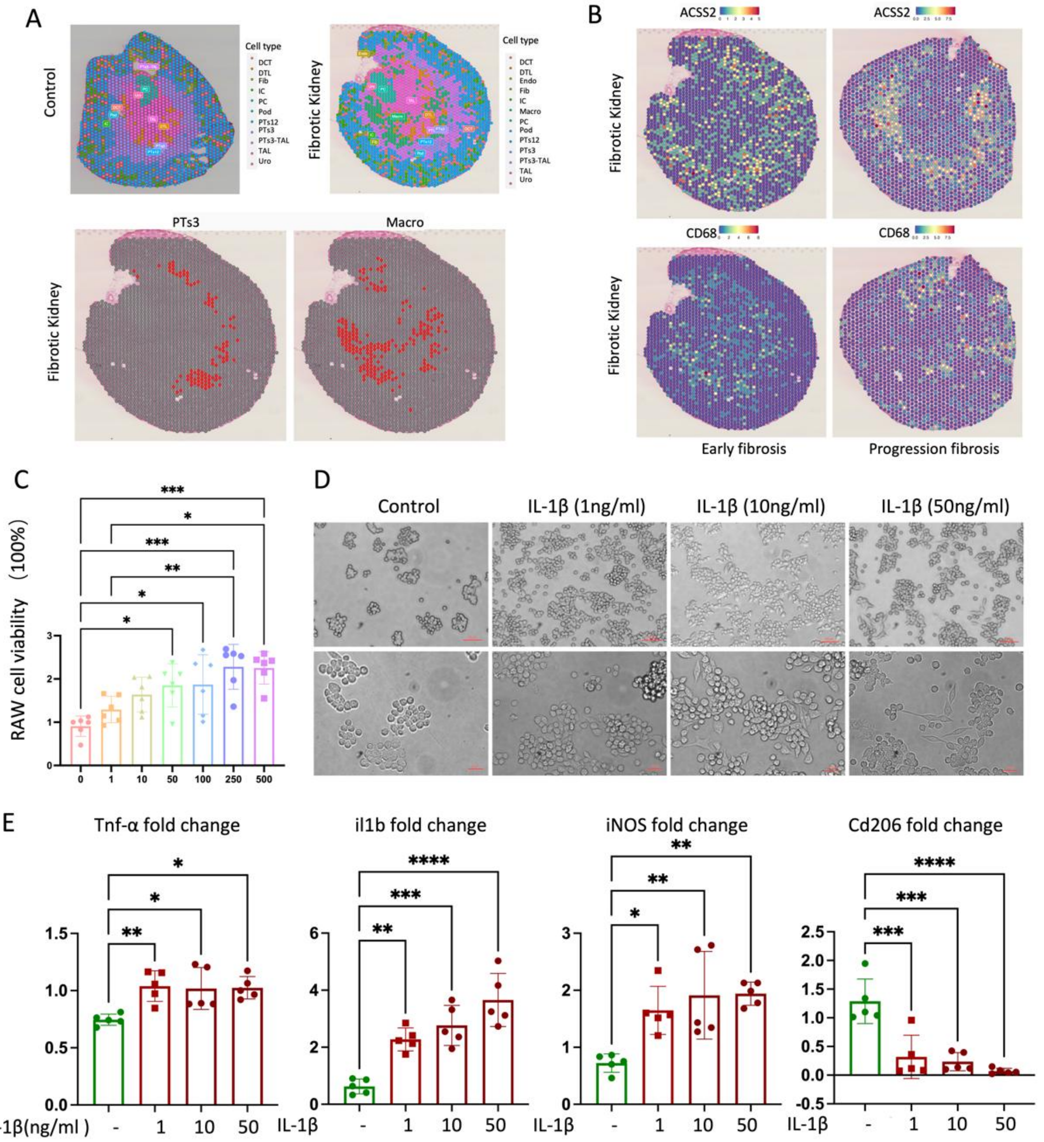

A
Control
Fibrotic Kidney
Cell type
DCT
DTL
Fib
IC
PC
Pod
PTs12
PTs3
PTs3-TAL
TAL
Uro
Endo
Macro
PTs3
Macro
B
ACSS2
CD68
Early fibrosis
Progression fibrosis
C
RAW cell viability （100%）
0
1
10
50
100
250
500
D
Control
IL-1β (1ng/ml)
IL-1β (10ng/ml)
IL-1β (50ng/ml)
E
Tnf-α fold change
il1b fold change
iNOS fold change
Cd206 fold change
IL-1β(ng/ml)
IL-1β

Supplementary Fig. 21. The changes of macrophage polarization when changing H3K9cr level by ACSS2 alteration both *in vitro* and *in vivo*. (A) The iNOS, Tnf-α, Mcp1, Il6, and IL-1β mRNA levels of RAW264.7 which was stimulated with cellular supernatants from HEK-293T cells transfected with ACSS2 plasmids for 24h (n = 3 per group). (B) The iNOS, Mcp1 and Tnf-α mRNA levels of RAW264.7 which was stimulated with cellular supernatants from TCMK-1 cells transfected with ACSS2 plasmids for 24 h (n = 3 per group). (C) Cd86 and Tnf-α mRNA levels in control and UUO of WT and $ACSS2^{-/-}$ mice (n = 5 per group). (D) Cd86 and Tnf-α mRNA levels in control and UUO of WT and $ACSS2^{tecKO}$ mice (n = 5 to 6 per group). (E) IF staining of F4/80 (yellow), iNOS (green), IL-1β (red) and DAPI (blue) in UUO kidneys of WT and $ACSS2^{-/-}$ mice. (F) IF staining of F4/80 (yellow), iNOS (green), IL1r1 (red) and DAPI (blue) in UUO kidneys of WT and $ACSS2^{-/-}$ mice. ACSS2OE: ACSS2 overexpression. WT: wild type; UUO: unilateral ureteric obstruction; $ACSS2^{-/-}$: ACSS2 knockout. Data shown are means ± SEM. Statistical analysis by one-way ANOVA with Tukey's post hoc test. *P < 0.05, **P < 0.01, ***P < 0.001 and ****P < 0.0001.

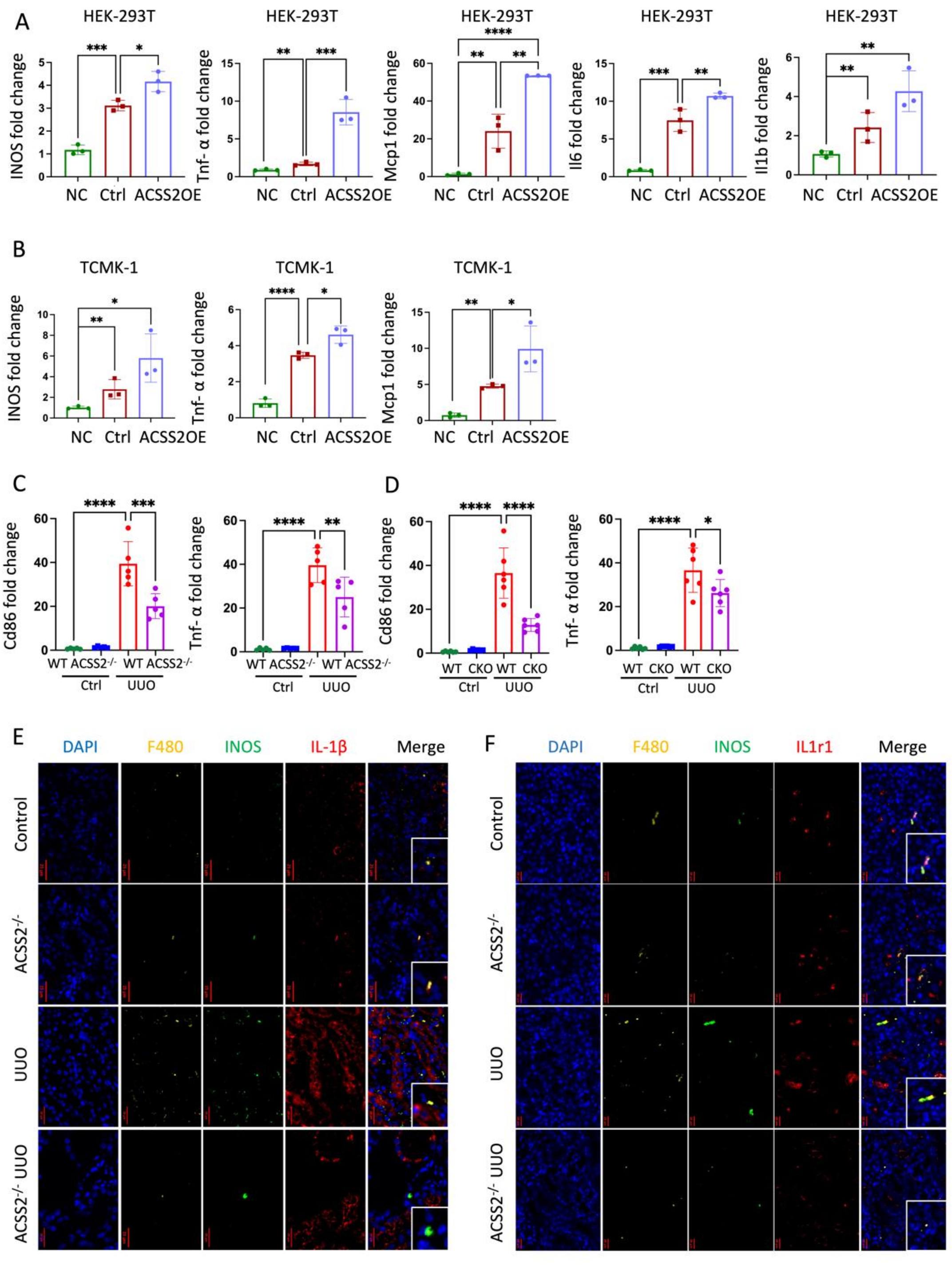

A
HEK-293T
INOS fold change
Tnf- α fold change
Mcp1 fold change
Il6 fold change
Il1b fold change
NC
Ctrl
ACSS2OE
B
TCMK-1
C
D
Cd86 fold change
WT
ACSS2-/-
CKO
Ctrl
UUO
E
F
DAPI
F480
INOS
IL-1β
IL1r1
Merge
Control
ACSS2-/-
UUO
ACSS2-/- UUO

Supplementary Fig. 22. The changes of macrophage polarization when changing H3K9cr level using HDAC1/2/3 and SIRT1/2/3 plasmids. (A) Microscopic images depicted the changes of RAW264.7 cells morphology after simulation of cellular supernatants from 293T cells transfected with HDAC1/2/3 and SIRT1/2/3 plasmids for 24 h (n = 3 per group).Scale bar: upper panels: 20 μm; lower panels:50 μm. (B) iNOS, Tnf-α and Mcp1 mRNA levels of RAW264.7 which was stimulated with cellular supernatants from TCMK-1 cells transfected with HDAC1/2/3 and SIRT1/2/3 plasmids for 24 h (n = 3 per group). (C) iNOS, Tnf-α, Mcp1, Il6, and IL-1β mRNA levels of RAW264.7 which was stimulated with cellular supernatants from HEK-293T cells transfected with HDAC1/2/3 and SIRT1/2/3 plasmids for 24 h (n = 3 per group). Data shown are means ± SEM. Statistical analysis by one-way ANOVA with Tukey's post hoc test. *$P < 0.05$, **$P < 0.01$, ***$P < 0.001$ and ****$P < 0.0001$.

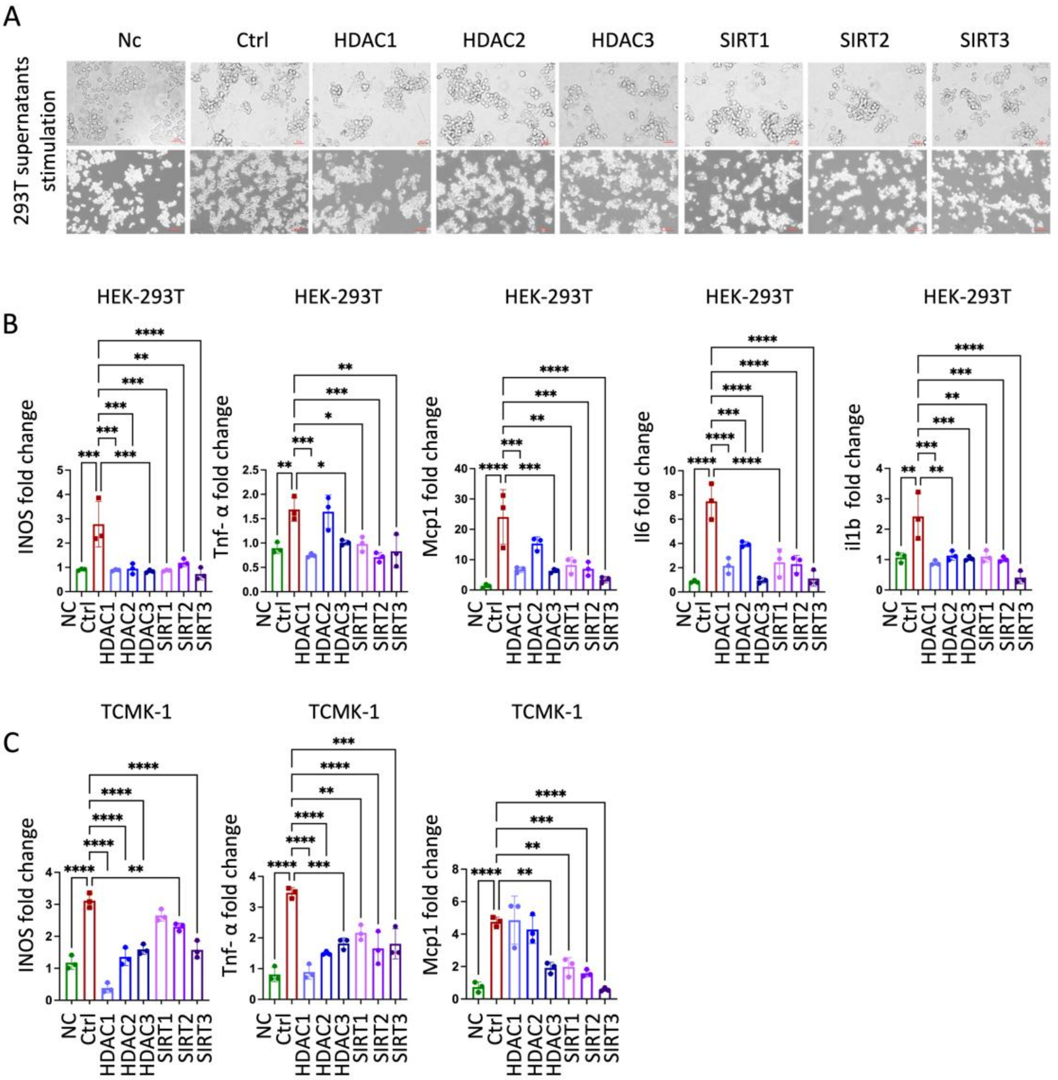
A
Nc
Ctrl
HDAC1
HDAC2
HDAC3
SIRT1
SIRT2
SIRT3
293T supernatants stimulation
B
HEK-293T
INOS fold change
Tnf- α fold change
Mcp1 fold change
Il6 fold change
il1b fold change
NC
Ctrl
HDAC1
HDAC2
HDAC3
SIRT1
SIRT2
SIRT3
C
TCMK-1
INOS fold change
Tnf- α fold change
Mcp1 fold change

Supplementary Fig. 23. The changes of cellular senescence when treating IL-1β in vitro. (A) CCK8 results of TCMK-1 cells stimulating with different doses of IL-1β (n = 6 per group). (B) SA-β-gal staining of primary tubular epithelial cells treated with IL-1β (5ng/ml) for 24h.Scale bar: 20 μm.(n= 2 per group). (C) P53, IL-1β, Il6 and Mmp9 mRNA levels of TCMK-1 cells which was stimulated with different doses of IL-1β (n = 3 per group). Data shown are means ± SEM. Statistical analysis by one-way ANOVA with Tukey's post hoc test. *P < 0.05, **P < 0.01, ***P < 0.001 and ****P < 0.0001.

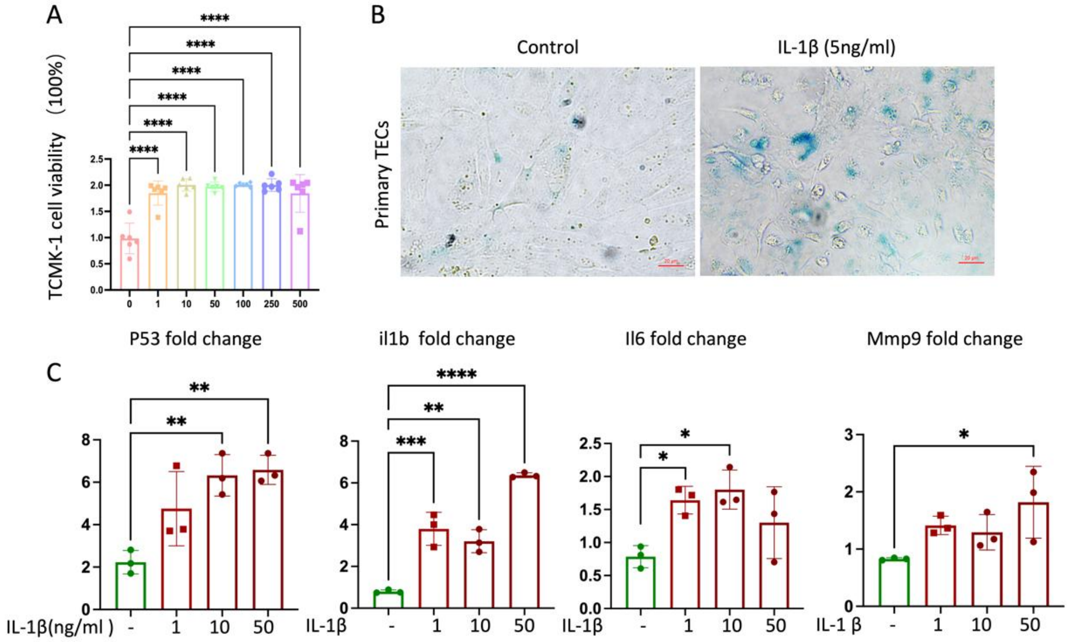

Supplementary Fig. 24. The changes of cellular senescence when changing H3K9cr level both in vivo and in vitro. (A) P53 and GAPDH immunoblotting in TCMK-1 cells which are stimulated by supernatants from TCMK-1 and HEK-293T cells transfected with ACSS2 plasmids (n = 3 per group). (B) P53, Mcp1 and Il6 mRNA levels of TCMK-1 cells stimulated with cellular supernatants from TCMK-1 cells transfected with ACSS2 plasmids. IL-1β antibody (5μg/ml) was added to neutralize supernatants (n = 6 per group). (C) P53, IL6, MCP1 and GAPDH immunoblotting in control and UUO mice (n = 5 to 6 per group). (D) P53, Il6 and Mcp1 mRNA levels of whole kidney lysates of control and UUO mice (n = 3 to 6 per group). (E) P53, IL6, MCP1 and GAPDH immunoblotting in control and FAN mice (n = 6 per group). (F) P53, Il6 and Mcp1 mRNA levels of whole kidney lysates of control and FAN mice (n = 3 to 6 per group). (G) P53, IL6, MCP1 and GAPDH immunoblotting in the whole kidney lysates of control and UUO of WT and $ACSS2^{-/-}$ mice (n = 3 per group). (H) P53, Il6 and Mcp1 mRNA levels in the whole kidney lysates of control and UUO of WT and $ACSS2^{-/-}$ mice (n = 5 per group). (I) P53, IL6, MCP1 and GAPDH immunoblotting in the whole kidney lysates of control and FAN of WT and $ACSS2^{-/-}$ mice (n = 3 per group). (J) P53, Il6 and Mcp1 mRNA levels in the whole kidney lysates of control and FAN of WT and $ACSS2^{-/-}$ mice (n = 3 to 5 per group). (K) P53, IL6, MCP1 and GAPDH immunoblotting in the whole kidney lysates of control and UUO of WT and $ACSS2^{tecKO}$ mice (n = 3 per group). (L) P53, Il6 and Mcp1 mRNA levels in the whole kidney lysates of control and UUO of WT and $ACSS2^{tecKO}$ mice (n = 5 to 6 per group). WT: wild type; FAN: folic acid

nephropathy; Ctrl: Control; UUO: unilateral ureteric obstruction; ACSS2$^{-/-}$: ACSS2 knockout; ACSS2 CKO: tubular epithelial cell-specific deletion of ACSS2. Data shown are means ± SEM. Statistical analysis by t-test or one-way ANOVA with Tukey's post hoc test. *P < 0.05, **P < 0.01, ***P < 0.001 and ****P < 0.0001.

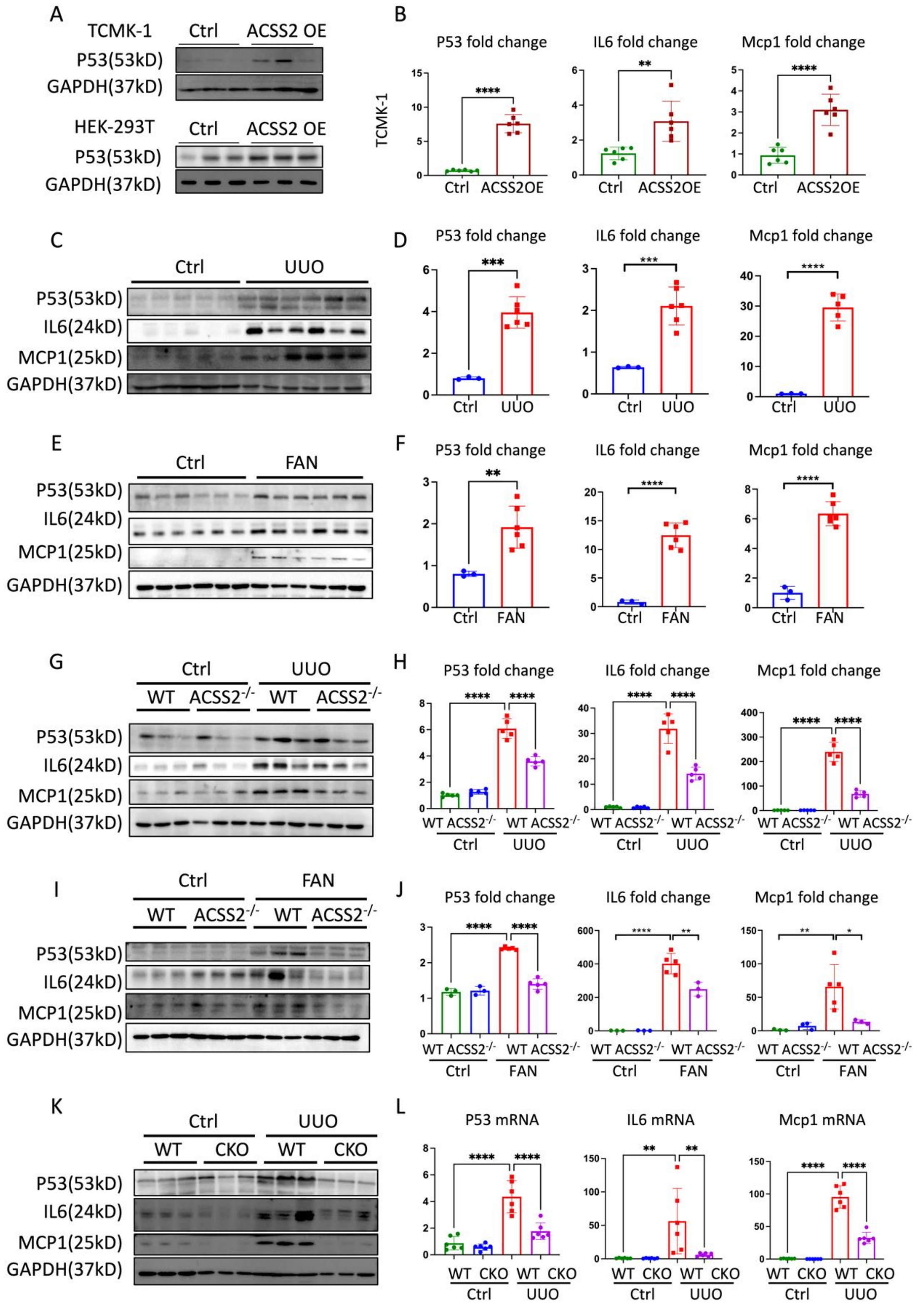
A
TCMK-1
Ctrl
ACSS2 OE
P53(53kD)
GAPDH(37kD)
HEK-293T
Ctrl
ACSS2 OE
P53(53kD)
GAPDH(37kD)
B
TCMK-1
P53 fold change
IL6 fold change
Mcp1 fold change
Ctrl
ACSS2OE
C
Ctrl
UUO
P53(53kD)
IL6(24kD)
MCP1(25kD)
GAPDH(37kD)
D
P53 fold change
IL6 fold change
Mcp1 fold change
Ctrl
UUO
E
Ctrl
FAN
P53(53kD)
IL6(24kD)
MCP1(25kD)
GAPDH(37kD)
F
P53 fold change
IL6 fold change
Mcp1 fold change
Ctrl
FAN
G
Ctrl
UUO
WT
ACSS2-/-
P53(53kD)
IL6(24kD)
MCP1(25kD)
GAPDH(37kD)
H
P53 fold change
IL6 fold change
Mcp1 fold change
WT ACSS2-/-
Ctrl
UUO
I
Ctrl
FAN
WT
ACSS2-/-
P53(53kD)
IL6(24kD)
MCP1(25kD)
GAPDH(37kD)
J
P53 fold change
IL6 fold change
Mcp1 fold change
WT ACSS2-/-
Ctrl
FAN
K
Ctrl
UUO
WT
CKO
P53(53kD)
IL6(24kD)
MCP1(25kD)
GAPDH(37kD)
L
P53 mRNA
IL6 mRNA
Mcp1 mRNA
WT CKO
Ctrl
UUO

Supplementary Fig. 25. Immunofluorescence staining of γH2AX when changing ACSS2 expression both *in vivo* and *in vitro*. (A) IF staining of γH2AX (green) and DAPI (blue) in UUO kidneys of WT and ACSS2$^{-/-}$ mice and treated with ACSS2 inhibitor. Scale bar: 25 μm. (n= 3 per group). (B) IF staining of γH2AX (green) and DAPI (blue) in PTC from WT and ACSS2$^{-/-}$ mice and HEK-293T cells transfected with ACSS2 plasmids tread with TGFβ1.Scale bar: 50 μm. (n= 3 per group). IF: immunofluorescence; PTC: primary tubular epithelial cells; WT: wild type; UUO: unilateral ureteric obstruction; ACSS2$^{-/}$-: ACSS2 knockout; ACSS2OE:ACSS2 overexpression.

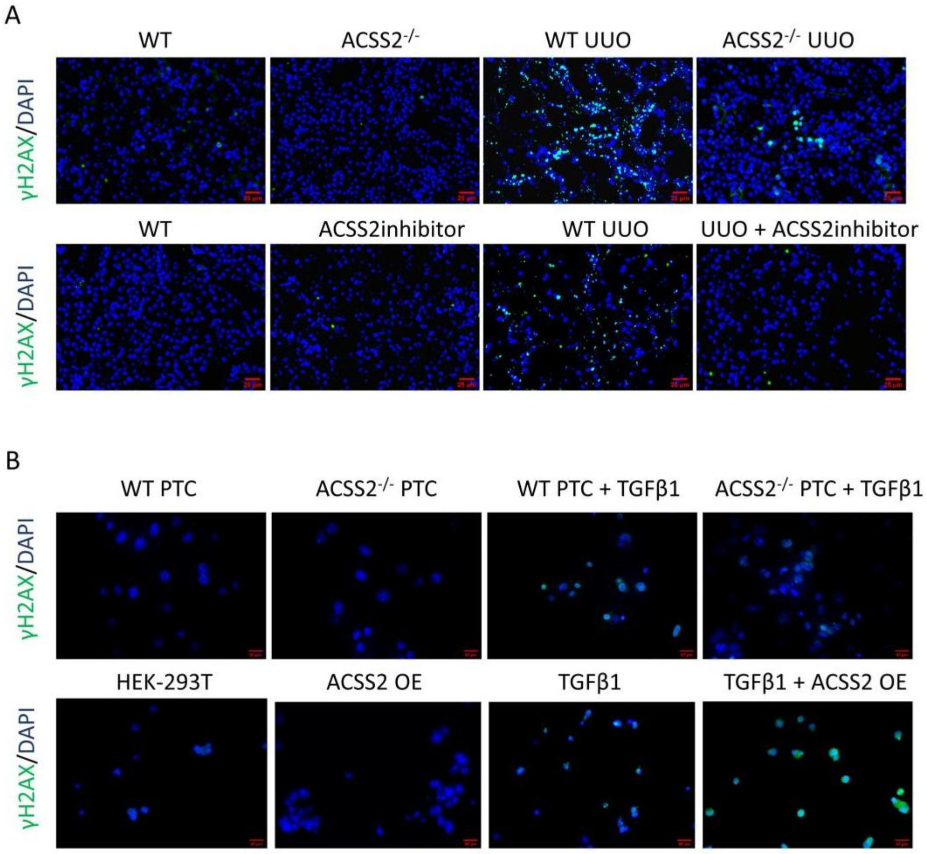

Supplementary Fig. 26. Anti-IL-1β IgG decreased kidney fibrosis, M1 macrophage and cellular senescence markers expression. (A) Quantification of COL6 and α-SMA immunoblots normalizing to GAPDH in the whole kidney lysates of control and UUO mice treated with IL-1β antibody (10 mg/kg) (n = 3 per group). (B) Quantification of IL-1β and IL6 immunoblots normalizing to GAPDH in the whole kidney lysates of control and UUO mice treated with IL-1β antibody (10 mg/kg) (n = 3 per group). (C) Quantification of P53 and MCP1 immunoblots normalizing to GAPDH in the whole kidney lysates of control and UUO mice treated with IL-1β antibody (10 mg/kg) (n = 3 per group). UUO: unilateral ureteric obstruction; IL-1β Ab: IL-1β neutralizing antibody. Data shown are means ± SEM. Statistical analysis by one-way ANOVA with Tukey's post hoc test. *P < 0.05, **P < 0.01, ***P < 0.001 and ****P < 0.0001.

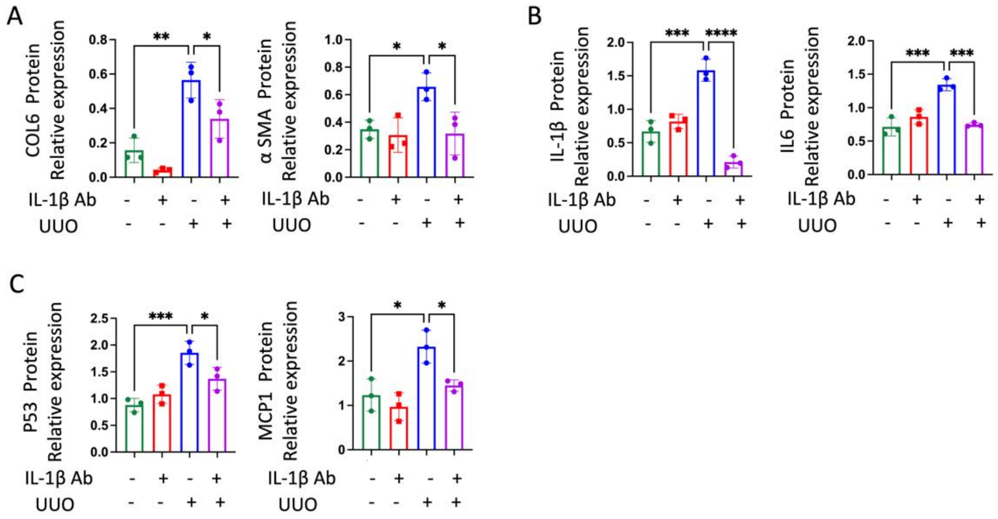

Supplementary Fig. 27. Recombinant IL-1β aggravated kidney fibrosis which was suppressed by ACSS2 inhibitor. (A-B) IL-1β, Col1a1 and Acta2 mRNA levels in the whole kidney lysates of control and UUO mice treated with recombinant IL-1β and ACSS2 inhibitor (n =6 per group). (C) IF staining and quantitative analysis of α-SMA (green) and DAPI (blue) in control and UUO mice treated with recombinant IL-1β and ACSS2 inhibitor. (n= 3 per group). Scale bar: 20 μm.(D) Masson staining and quantitative analysis in control and UUO mice treated with recombinant IL-1β and ACSS2 inhibitor. (n= 3 per group). Scale bar: 50 μm. UUO: unilateral ureteric obstruction; ACSS2in: ACSS2 inhibitor. Data shown are means ± SEM. Statistical analysis by one-way ANOVA with Tukey's post hoc test. $^{*}P < 0.05$, $^{**}P < 0.01$, $^{***}P < 0.001$ and $^{****}P < 0.0001$.

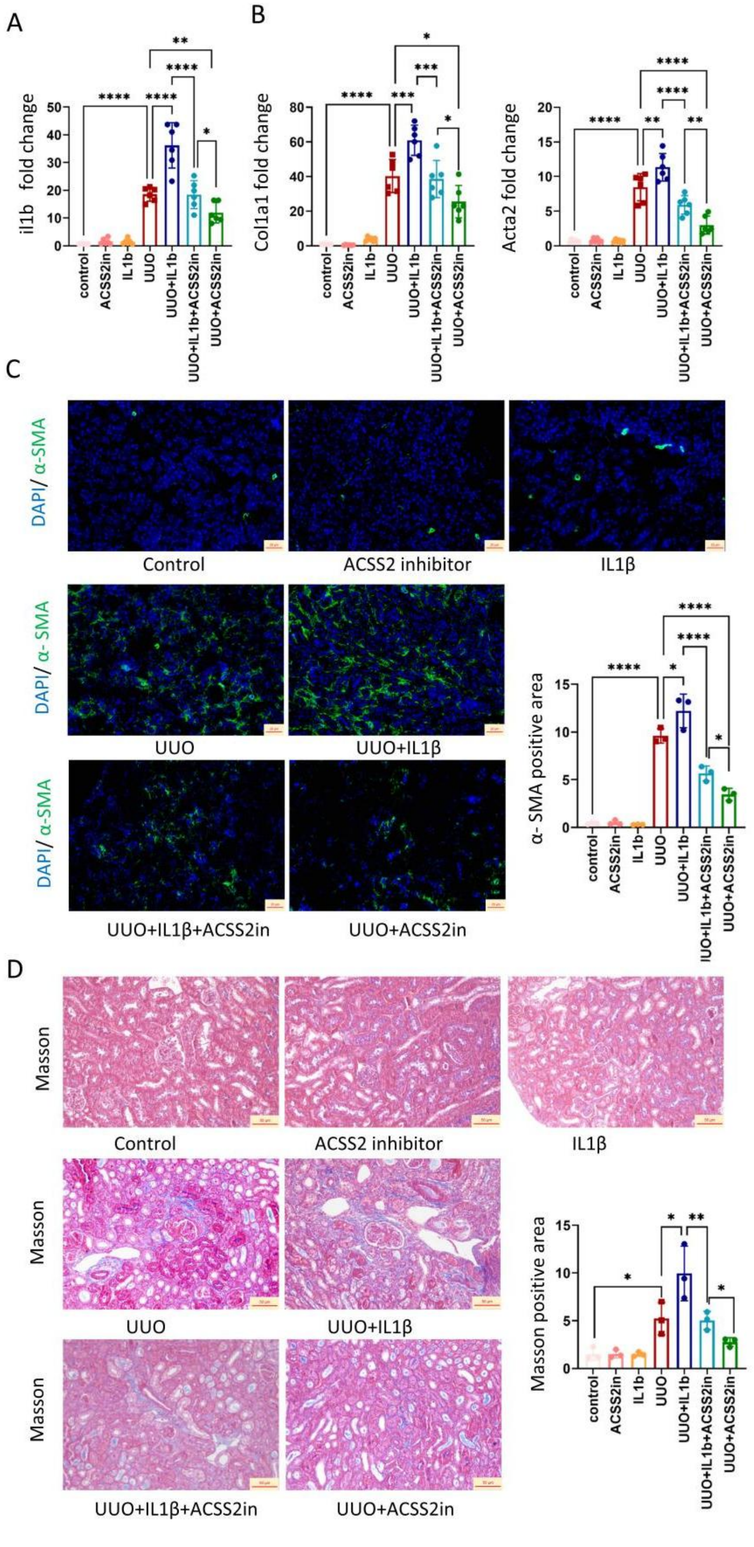
A
B
il1b fold change
Col1a1 fold change
Acta2 fold change
control
ACSS2in
IL1b
UUO
UUO+IL1b
UUO+IL1b+ACSS2in
UUO+ACSS2in
C
DAPI/ α-SMA
Control
ACSS2 inhibitor
IL1β
UUO
UUO+IL1β
UUO+IL1β+ACSS2in
UUO+ACSS2in
α- SMA positive area
D
Masson
Masson positive area

Supplementary Fig. 28. Recombinant IL-1β aggravated cellular senescence in fibrotic kidneys of UUO mice which was suppressed by ACSS2 inhibitor. (A) SA-β-gal staining in kidneys of control and UUO mice treated with recombinant IL-1β and ACSS2 inhibitor. Scale bar: 25 μm. (n= 3 per group). Scale bar: 20 μm.(B) Quantitative analysis of SA-β-gal staining in kidneys of control and UUO mice treated with recombinant IL-1β and ACSS2 inhibitor. (C) TNFα, CD86 and MMP9 mRNA levels in the whole kidney lysates of control and UUO mice treated with recombinant IL-1β and ACSS2 inhibitor (n = 6 per group). (D) IF staining of γH2AX (green) and DAPI (blue) in control and UUO mice treated with recombinant IL-1β and ACSS2 inhibitor. Scale bar: 20 μm. (n= 3 per group). UUO: unilateral ureteric obstruction; ACSS2in: ACSS2 inhibitor. Data shown are means ± SEM. Statistical analysis by one-way ANOVA with Tukey's post hoc test. *P < 0.05, **P < 0.01, ***P < 0.001 and ****P < 0.0001.

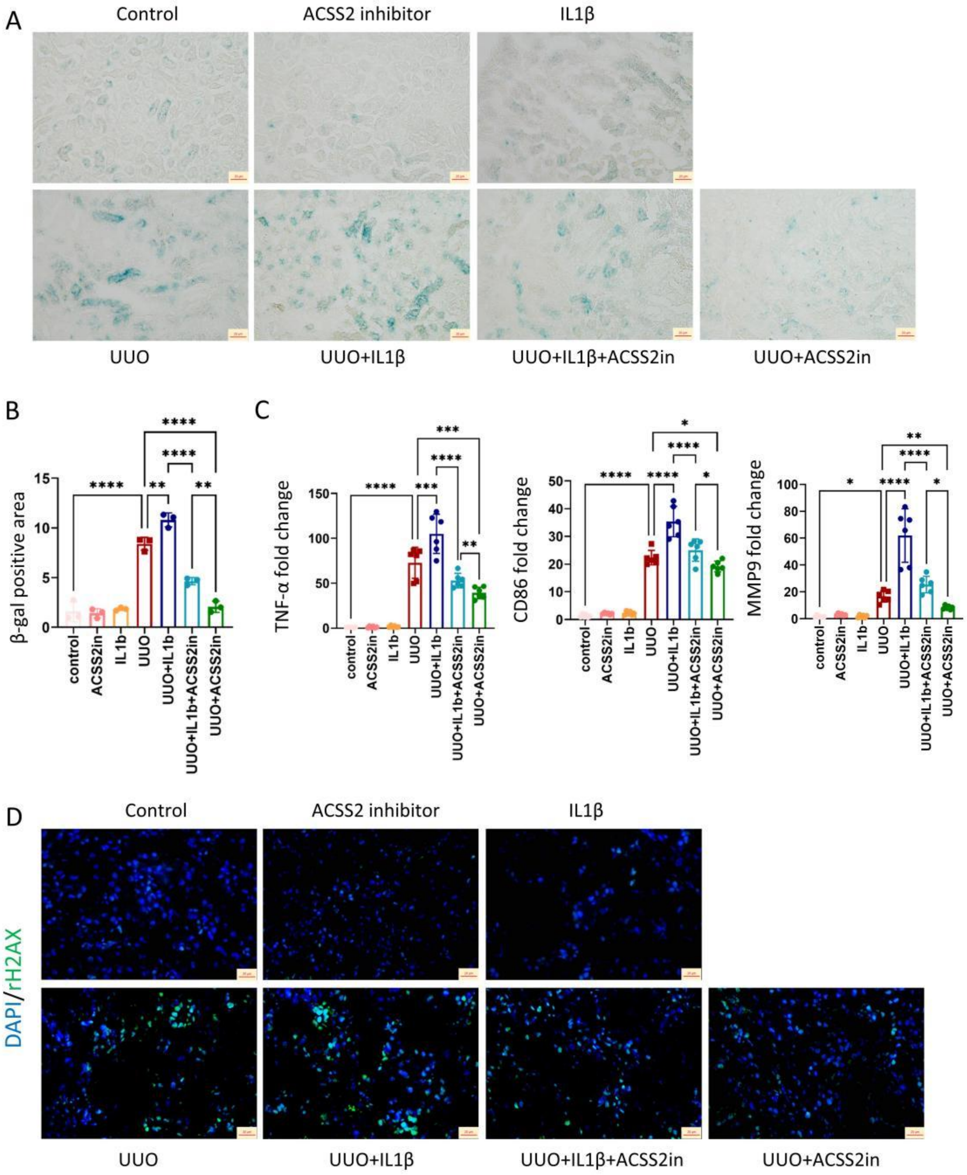
A
Control
ACSS2 inhibitor
IL1β
UUO
UUO+IL1β
UUO+IL1β+ACSS2in
UUO+ACSS2in
B
β-gal positive area
control
ACSS2in
IL1b
UUO
UUO+IL1b
UUO+IL1b+ACSS2in
UUO+ACSS2in
C
TNF-α fold change
CD86 fold change
MMP9 fold change
D
DAPI/rH2AX
Control
ACSS2 inhibitor
IL1β
UUO
UUO+IL1β
UUO+IL1β+ACSS2in
UUO+ACSS2in

Supplementary Fig. 29. The assessment of ACSS2 inhibitor in mice. (A) The BUN, Scr, ALT and AST level in control and ACSS2 inhibitor-treated mice. (B) The H&E staining for heart, liver, spleen, lung, kidney in control and ACSS2 inhibitor-treated group. (n= 2 per group). Scale bar: upper panels: 200 μm; lower panels:100 μm. Scr: serum creatinine, BUN: blood urea nitrogen, ALT: glutamic pyruvic transaminase, AST: glutamic oxaloacetic transaminase. Data shown are means ± SEM. Statistical analysis by t-test.

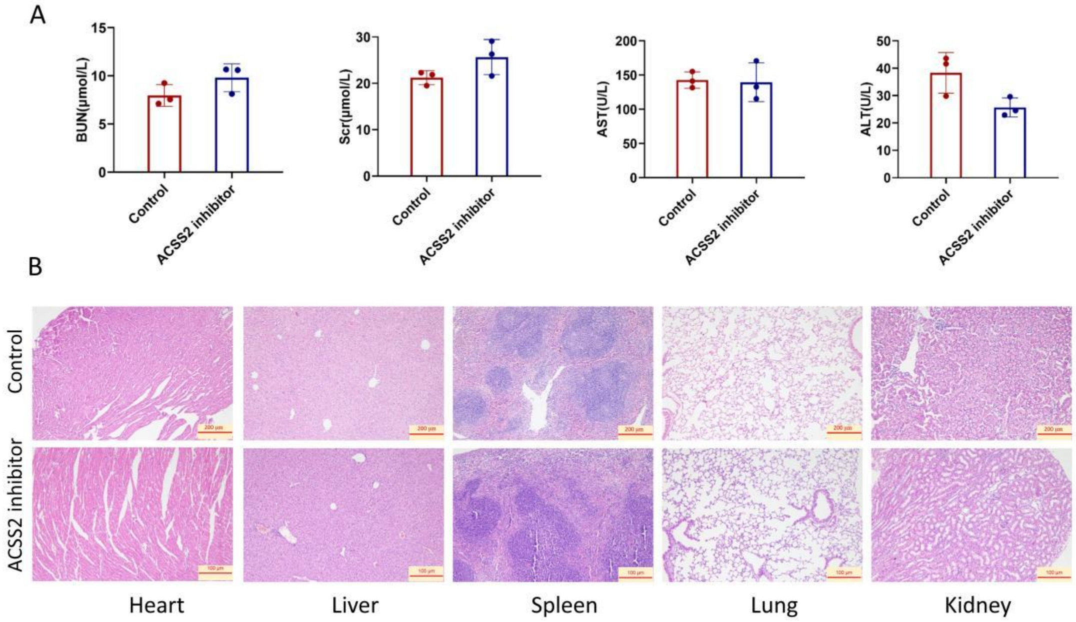

Supplementary Fig. 30. ACSS2 inhibitor suppressed H3K9cr level and alleviated kidney fibrosis in UUO and FAN models. (A) Protein expression of ACSS2 and GAPDH in whole kidney lysates of control and UUO/FAN fibrotic mice with or without ACSS2 inhibitor treatment (n = 3 per group). (B-C) Protein level of H3K9cr, H3K9ac and H3 and quantification of these immunoblots in whole kidney lysates of FAN mice treated with or without ACSS2 inhibitor (n = 3 per group). (D) FN1, COL6, α-SMA and GAPDH immunoblotting in the whole kidney lysates of FAN mice treated with or without ACSS2 inhibitor (n = 3 per group). (E) mRNA levels of Fn1, Col1a1, Acta2 and Kim1 in whole kidney lysates of control, UUO fibrotic mice and UUO mice treated with ACSS2 inhibitor (n = 4 to 6 per group). (F) Representative images of H&E and Masson staining in FAN mice treated with ACSS2 inhibitor. Scale bar: 100 μm. (n= 3 per group). (G) Quantitative analysis of COL6, FN1, α-SMA in UUO mice treated with ACSS2 inhibitor. Ctrl: Control; FAN: folic acid nephropathy; UUO: unilateral ureteric obstruction. Data shown are means ± SEM. Statistical analysis by one-way ANOVA with Tukey's post hoc test. *P < 0.05, **P < 0.01, ***P < 0.001 and ****P < 0.0001.

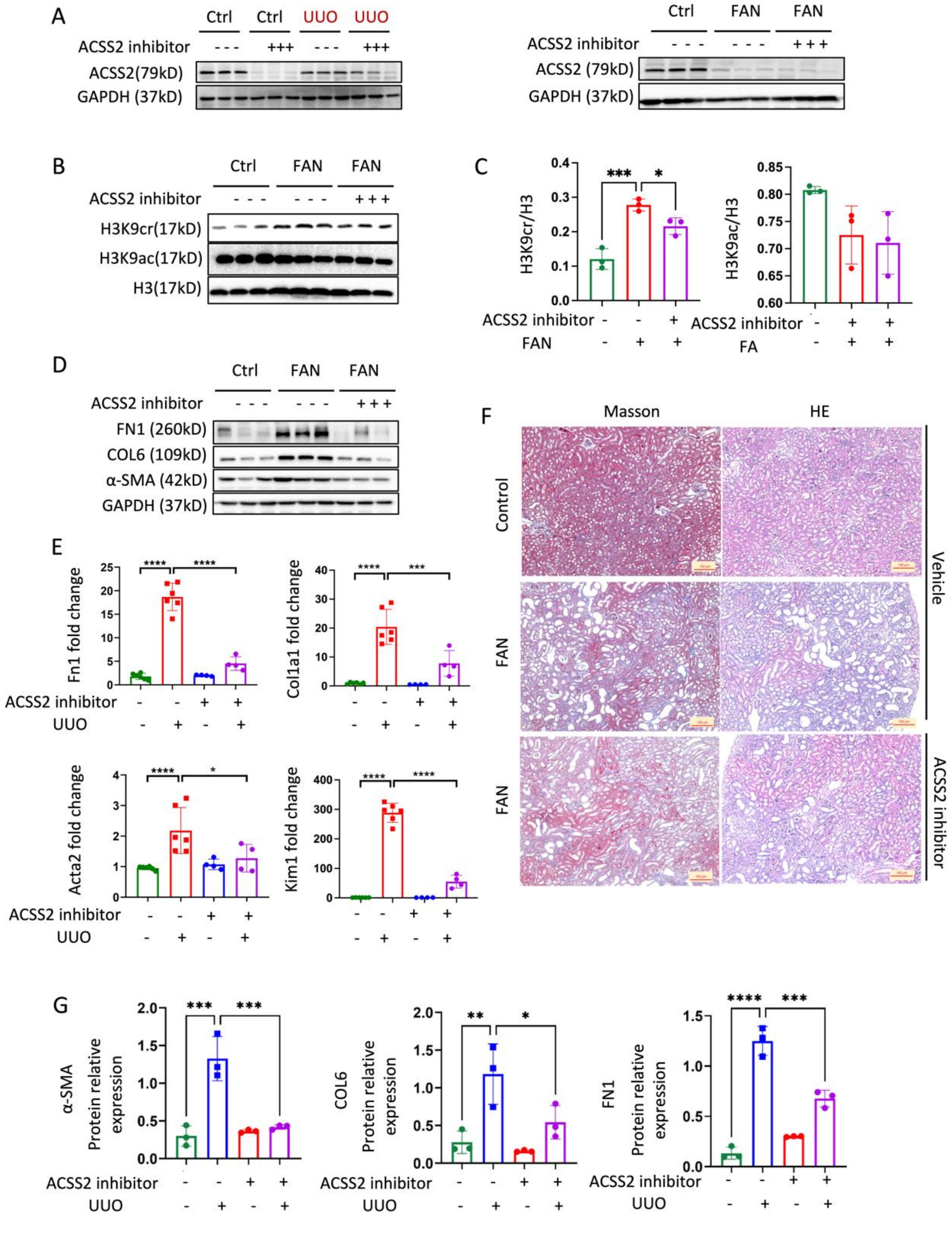

A
Ctrl
Ctrl
UUO
UUO
ACSS2 inhibitor
- - -
+++
ACSS2(79kD)
GAPDH (37kD)
Ctrl
FAN
FAN
ACSS2 (79kD)
GAPDH (37kD)
B
H3K9cr(17kD)
H3K9ac(17kD)
H3(17kD)
C
H3K9cr/H3
H3K9ac/H3
FAN
FA
D
FN1 (260kD)
COL6 (109kD)
α-SMA (42kD)
GAPDH (37kD)
E
Fn1 fold change
Col1a1 fold change
Acta2 fold change
Kim1 fold change
F
Masson
HE
Control
FAN
Vehicle
ACSS2 inhibitor
G
α-SMA
COL6
FN1
Protein relative expression

Supplementary Fig. 31. ACSS2 inhibitor suppressed H3K9cr-mediated IL-1β expression, decreased cellular senescence and M1 macrophage markers in UUO and FAN models. (A) Protein expression of IL-1β, cleaved-IL-1β and GAPDH in whole kidney lysates of control and UUO mice with or without ACSS2 inhibitor treatment (n = 3 per group). (B) Quantification of IL-1β and cleaved-IL-1β immunoblots normalizing to GAPDH in whole kidney lysates of control and UUO mice with or without ACSS2 inhibitor treatment (n = 3 per group). (C) Protein expression of IL-1β, cleaved-IL-1β and GAPDH in whole kidney lysates of control and FAN mice with or without ACSS2 inhibitor treatment (n = 3 per group). (D) Quantification of IL-1β immunoblots and cleaved-IL-1β normalizing to GAPDH as well as mRNA levels of Il1b in whole kidney lysates of control and UUO mice with or without ACSS2 inhibitor treatment (n = 3 to 6 per group). (E) SA-β-gal staining of control, FAN fibrotic mice and FAN mice treated with ACSS2 inhibitor. (n= 2 per group). (F) mRNA levels of P53 and Il6 in whole kidney lysates of control, FA fibrotic mice and FA mice treated with ACSS2 inhibitor (n = 3 to 6 per group). Scale bar: upper panels: 200 μm; lower panels:100 μm. (G) Protein expression of P53, MCP1, IL6 and GAPDH in whole kidney lysates of control, FA fibrotic mice and FA mice treated with ACSS2 inhibitor (n = 3 per group). (H) Quantification of P53, MCP1 and IL6 immunoblots normalizing to GAPDH in whole kidney lysates of control, FA fibrotic mice and FA mice treated with ACSS2 inhibitor (n = 3 per group). (I) Protein expression of P53, IL6 and GAPDH in whole kidney lysates of control and UUO mice with or without ACSS2 inhibitor treatment (n = 3 per group). (J)

Quantification of P53 and IL6 immunoblots normalizing to GAPDH in whole kidney lysates of control, FA fibrotic mice and FA mice treated with ACSS2 inhibitor ($n = 3$ per group). Ctrl: Control; FAN: folic acid nephropathy; UUO: unilateral ureteric obstruction. Data shown are means ± SEM. Statistical analysis by one-way ANOVA with Tukey's post hoc test. *$P < 0.05$, **$P < 0.01$, ***$P < 0.001$ and ****$P < 0.0001$.

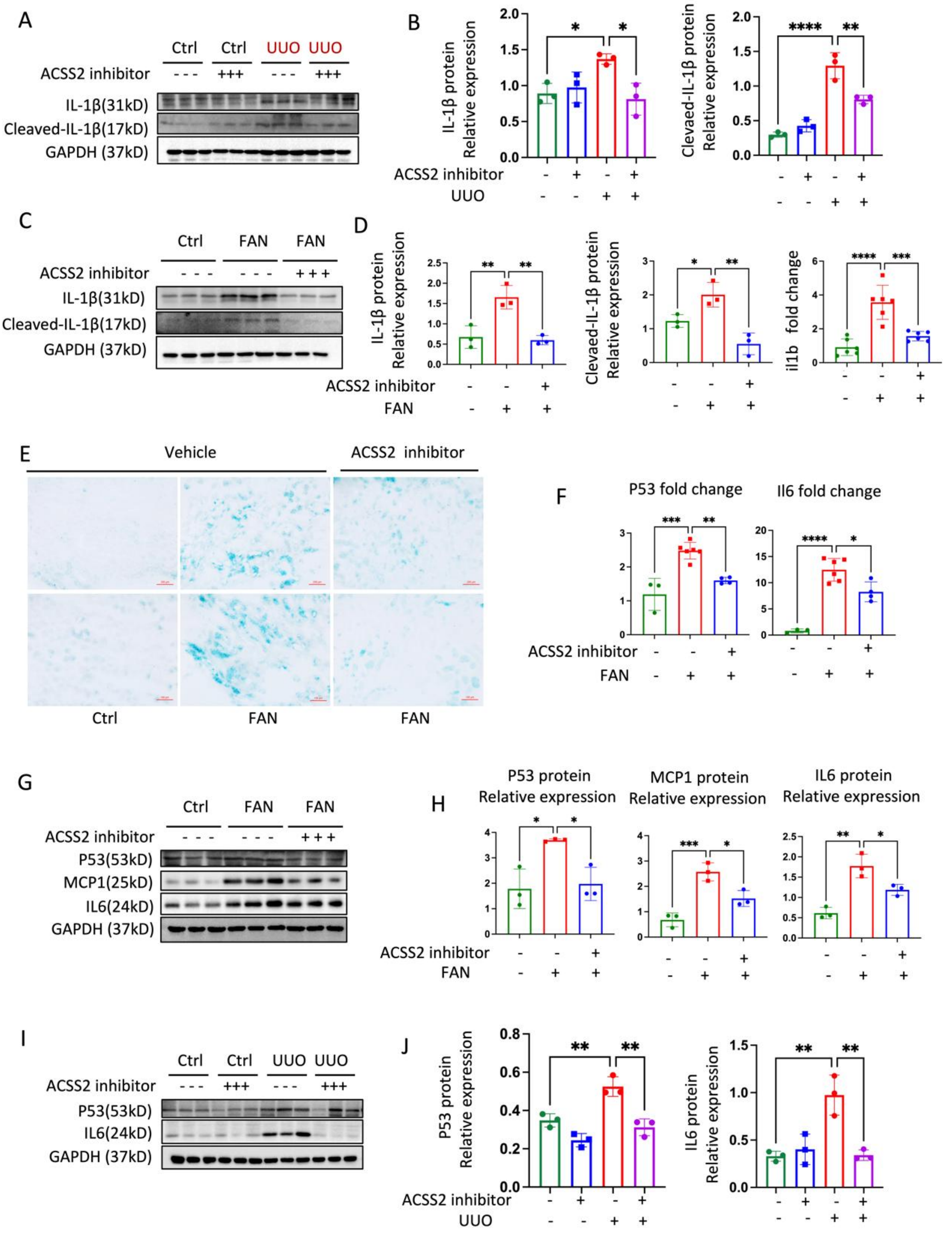
A
Ctrl
Ctrl
UUO
UUO
ACSS2 inhibitor
- - -
+++
- - -
+++
IL-1β(31kD)
Cleaved-IL-1β(17kD)
GAPDH (37kD)
B
IL-1β protein
Relative expression
Clevaed-IL-1β protein
Relative expression
ACSS2 inhibitor
UUO
C
Ctrl
FAN
FAN
ACSS2 inhibitor
IL-1β(31kD)
Cleaved-IL-1β(17kD)
GAPDH (37kD)
D
IL-1β protein
Relative expression
Clevaed-IL-1β protein
Relative expression
il1b fold change
ACSS2 inhibitor
FAN
E
Vehicle
ACSS2 inhibitor
Ctrl
FAN
FAN
F
P53 fold change
Il6 fold change
ACSS2 inhibitor
FAN
G
Ctrl
FAN
FAN
ACSS2 inhibitor
P53(53kD)
MCP1(25kD)
IL6(24kD)
GAPDH (37kD)
H
P53 protein
Relative expression
MCP1 protein
Relative expression
IL6 protein
Relative expression
ACSS2 inhibitor
FAN
I
Ctrl
Ctrl
UUO
UUO
ACSS2 inhibitor
P53(53kD)
IL6(24kD)
GAPDH (37kD)
J
P53 protein
Relative expression
IL6 protein
Relative expression
ACSS2 inhibitor
UUO
