# Supplementary material for: Inhibition of ACSS2-mediated histone crotonylation alleviates kidney fibrosis via IL-1β-dependent macrophage activation and tubular cell senescence
Source: Nat Commun. 2024 Apr 13;15:3200. doi: 10.1038/s41467-024-47315-3 (PMC11016098; doi:10.1038/s41467-024-47315-3)
Supplement: Supplementary file 3 — Description of Additional Supplementary Files [file 41467_2024_47315_MOESM3_ESM.pdf]

Description of Additional Supplementary Files

Supplementary Data 1 Description: The basis characteristics of the patients in figure 1A.
Data presented as mean ± SD. CKD-EPI, Chronic Kidney Disease Epidemiology Collaboration; ANCA, Anti-Neutrophil Cytoplasmic Antibodies.

Supplementary Data 2 Description: Multivariate regression of patients to adjust age and sex influence in figure1B
Multivariate regression adjusting age and sex. *p<0.05, **p<0.01, ***p<0.001, ****p<0.0001, ns: no significant. Two-sided statistical tests.

Supplementary Data 3 Description: The pharmacokinetic data of ACSS2 inhibitor via i.p. administration.
AUC, area under the plasma concentration-time curve; $T_{1/2}$, half-time; T_{max}, time taken to reach maximum plasma concentration; C_{max}, maximum plasma concentration.

Supplementary Data 4 Description: The list of chemicals and primary antibodies.
Information (company and catalog number) of the chemicals and primary antibodies used in our manuscripts.

Supplementary Data 5 Description: The list of primer sequences.
The sequence of all the primers used in our manuscript.
